# Dissociable effects of fatigue on performance and metacognition from automatic target cuing in undersea threat detection

**DOI:** 10.1186/s41235-025-00638-1

**Published:** 2025-06-15

**Authors:** Max Kailler Smith, Amelia R. Kracinovich, Brandon J. Schrom, Timothy L. Dunn

**Affiliations:** 1 Warfighter Performance Department, Naval Health Research Center, San Diego, CA USA; 2 Leidos, Inc., San Diego, CA USA; 3 Naval Information Warfare Center Pacific, San Diego, CA USA

**Keywords:** Human–automation interaction, Fatigue, Vigilance, Visual search, Metacognition, Trust in automation

## Abstract

As automation becomes increasingly integrated into complex military tasks, its role in supporting human performance under fatigue warrants careful evaluation. A specific military use case in which automatic target cuing (ATC) is integrated is undersea threat detection (UTD). These types of tasks demand sustained vigilance, accurate classification, and reliable metacognitive judgements. Fatigue, especially due to increased time awake, presents a significant challenge to sustaining high performance. This study investigated whether ATC enhances UTD performance under low fatigue conditions and protects against errors when operators are fatigued, as is common during fleet operations. Thirty-six active-duty service members completed four sessions of a simulated UTD task, with and without an imperfect ATC system, over a ~24-hour wakeful period. Results showed that ATC did not enhance performance when participants were alert, though detection accuracy maintained despite increased fatigue. However, fatigue led to decreased metacognitive sensitivity, reflected in greater confidence for false alarms and reduced trust in the ATC system. These findings suggest that while automation assistance can potentially protect basic task performance under fatigue, it does not prevent the degradation of higher-level cognitive processes, such as metacognitive accuracy and trust in the automation. This study highlights the importance of understanding how automation interacts with cognitive states, especially under fatigue, to optimize its role in critical military operations.

**Supplementary Information:**

The online version contains supplementary material available at 10.1186/s41235-025-00638-1.

## Significance statement

Undersea threat detection (UTD) involves detecting and classifying potential threats on the ocean floor. UTD missions are often prolonged requiring naval operators to maintain sustained attention and make accurate judgments despite increased fatigue. Low level of automation technologies, such as automatic target cuing (ATC) systems, has the potential to support operator performance by visually highlighting possible target locations. However, it remains unclear how such systems influence both detection performance and metacognitive processes like detection confidence and trust in the ATC, especially under conditions of heightened fatigue. To investigate this, we conducted a study where active-duty personnel performed simulated UTD tasks with and without an ATC system over a 24-hour period, modeling real-world fatigue demands. We found that while detection accuracy remained stable across sessions with ATC despite increased fatigue, operators' metacognitive sensitivity and trust in the ATC suffered with increased time awake. These findings suggest that although performance was maintained under fatigue, higher-order cognitive processes, which underly complex decision-making in extended operations, were still vulnerable to fatigue effects. The results of this study highlight important limitations to consider when integrating even low level of automation assistive technologies into high-stakes task workflows, underscoring the need for further research into how attention, cognition, and metacognition are influenced by the use of imperfect automation, especially under fatigue. Insights from this study contribute to advancing military training and operational design, offering guidance for developing human–machine systems that more effectively support resilient performance across diverse, high-demand settings.

## Introduction

The ability to accurately perform complex cognitive tasks over prolonged periods, at any time of day, is mission critical in several military occupations. For example, the visual search for threatening targets is often performed during sustained operations and cognitive lapses can have lethal consequences. Platforms designed to assist service members with the detection, classification, and neutralization of these threats have been developed with technological advancements in automation. Automated solutions have ranged from low levels of automation (LOA) (e.g., the automation executes preprogrammed tasks or routines to offer suggestions or narrow decision/search space) to high levels of automation/autonomy (e.g., the computer has greater control over decision-making and execution processes and is not required to inform the human of its actions) (see O’Neil et al., 2022, and Parasuraman et al., 2000 for discussion of LOA criterion). One specific military context which stands to benefit by integrating such automation technologies is undersea threat detection (UTD).

UTD requires visual inspection of ocean floor data collected by unmanned vehicles to identify potential threats and other man-made objects of interest. In a typical UTD mission, operators must examine vast amounts of sonar data, knowing that threats may be extremely rare—it is not uncommon to inspect entire datasets without encountering any threats. Operators frequently perform these tasks under conditions of heightened physical and cognitive fatigue, poor sleep, and stress. In addition to these inherent operational stressors, UTD presents further challenges as search areas (the geographic regions of ocean floor that must be inspected) can be extensive, requiring sustained vigilance despite the low probability of encountering threats. This combination of extensive search and low target prevalence creates a particularly challenging task. Despite these challenging conditions, accurate detection and classification are paramount since minor detection errors and misses can result in catastrophes. The consequences of detection failures in UTD extend beyond immediate mission outcomes, potentially impacting broader naval operations, fleet safety, and strategic military objectives.

The present study investigated how automatic target cueing (ATC) affects operator performance and metacognitive processes during UTD under conditions of heightened fatigue. While ATC systems show promise for supporting detection performance, their effectiveness when operators are fatigued remains unclear. Of particular interest is how increased time awake affects not only basic detection performance with ATC assistance, but also higher-order cognitive processes such as confidence judgments and trust in the automation. Understanding these relationships is crucial for optimizing the integration of automation technologies into military operations where sustained performance under fatigue is mission critical.

### Effects of Automation on Performance and Behavior

Understanding how automation affects human performance and behavior requires first establishing what constitutes automation. Here, we adopt Parasuraman’s et al., (2000) definition of automation, which is “a device or system that accomplishes a function that was previously, or conceivably could be, carried out by a human operator” (pg. 286). When integrating automation into task workflows, it is essential to understand how automation behaviors, operator use behaviors, and metacognitive judgments about task performance interact and influence each other. Characterizing these interaction patterns has implications not just for UTD, but for the growing integration of automation across military operations where sustained human performance is critical. This interconnectedness means that these factors must be considered in relation to one another to identify and address how they impact task performance. To assist operators with UTD, a low-moderate LOA ATC system has been integrated, which highlights regions in a search space that likely contain a target object. That is, the ATC provides visual cues to assist efficient orienting to regions of interest during visual search. Though the ATC merely provides simple visual cues, previous research indicates that even for simple automation systems with limited capabilities, factors such as lack of transparency regarding system reliability can produce complex human–automation interactions (e.g., trust, reliance, compliance issues) that impact utilization and overall performance (Dixon & Wickens, 2006; Dzindolet et al., 2003; Woods, 2018). In addition to evaluating overall performance, it is therefore equally important to examine how operators use the automation. Calibrated use occurs when operator reliance appropriately matches the system's actual capabilities and limitations (Lee & See, 2004). By identifying conditions that disrupt calibrated use, we can better understand and prevent patterns of misuse and disuse.

Parasuraman (1997) discusses suboptimal use behaviors, including misuse and disuse. Misuse is defined as unwarranted overreliance on automation when it should not be used, whereas disuse is defined as underutilization of automation that should be used. Mosier and Skitka (1996) have shown that overreliance on automation can reduce attention to contradictory sources of information, leading individuals to ignore information that disconfirms automation suggestions. In the context of UTD, misuse of ATC that is prone to cue false positives could lead operators to ignore evidence of invalidly cued non-target objects, potentially resulting in incorrect threat assessments. Conversely, disuse occurs when operators perceive the automation as unreliable or overburdensome, leading them to ignore its cues entirely.

While maximizing correct detections by accepting a higher false alarm rate might seem optimal, including in UTD, an overly liberal detection threshold can backfire. The burden of verifying the numerous false positives can increase workload and promote disuse as operators become overwhelmed and distrustful. This “cry wolf” effect (Breznitz, 2013; Parasuraman & Riley, 1997) has been demonstrated in various contexts. For example, Dixon & Wickens (2006) found that compliance with automated alerts (i.e., fast and accurate responses to alerts) decreased when the system had a high false alarm rate during a simulated unmanned aerial vehicle task. The salience of automation errors also affects trust and utilization. Madhavan et al. (2006) found that when an automated aid (i.e., 20% miss rate and 40% false alarm rate) made errors on easily detectable targets, operators showed reduced trust and reliance, even when the system performed perfectly on difficult trials. These findings suggest that automation errors perceived to be obvious or easily avoidable are particularly damaging to operator trust and subsequent system use.

Building on the implications of misuse and disuse of automation, it is important to consider how the LOA and target prevalence—the number of targets actually present in a given search area—further shape operator performance and decision-making. Evidence suggests that low-moderate LOA, such as ATC which provides visual cues to potential target locations to narrow the search space, can benefit attention (Chavaillaz et al., 2018) and detection performance in military contexts (Rovira et al., 2007) despite the system being imperfect (de Visser & Parasuraman, 2011). Alternatively, overreliance on an ATC runs the risk of overlooking unexpected targets or succumbing to the *satisfaction of search* effect (i.e., terminating search when a target has been found) (Wolfe, 2012; Yeh, Wickens & Seagull, 1999). That is, the presence of an ATC detection may lead an operator to abandon continuation of search because they have determined they found the target or assume that the ATC must have identified all the potential targets. Target prevalence has also been shown to determine search termination threshold (Chun & Wolfe, 1996; Wolfe et al., 2007; Wolfe & Van Wert, 2010), with lower target prevalence increasing the likelihood of premature search termination (Ethell & Manning, 2001; Godwin et al., 2010; Wolfe, Horowitz, & Kenner, 2005). This relationship is particularly critical for UTD tasks, where target prevalence is sometimes commonly low. The challenge of maintaining vigilance when threats are rare, combined with the tendency toward early search termination in low-prevalence conditions, makes UTD especially vulnerable to greater error-likelihood, which can have life or death consequences.

Beyond the impact of automation behaviors and target prevalence on performance, understanding the role of human metacognitive processes, including subjective trust in automation (Hoff & Bashir, 2015; Lee & See, 2004) and system familiarity (O’Neill et al., 2022; Sanchez et al., 2014), is vital for optimizing operator engagement with automation systems. Engineering efforts tend to overlook metacognitive beliefs about automation (e.g., pre-existing beliefs about the system and trust in the system), which may consequently influence the way operators interact with systems. Automation solutions are frequently deployed with minimal military user input, who consequently gain little knowledge about the system’s features, purpose, and capabilities that influence operator trust (Schaefer et al., 2016). Research has demonstrated operator trust is strongly mediated by a system’s consistency, reliability, and transparency over time (Fan et al., 2008; McLeod et al., 2005). Trust increases when the operator has authority over system functioning (Sauer, Nickel, & Wastell, 2013), in addition to the appropriateness and type of visual cues (Muir & Moray, 1996; Wiegmann et al., 2006), the availability of information (Bitan & Meyer, 2007), and feedback accuracy Sharples, 2009).

For UTD, operator trust in the ATC system influences decision-making processes, including metacognitive judgements which are an integral component of task performance. In addition to basic detection and classification of threats, operators must provide standardized confidence ratings for their detection and classification calls, a requirement that reflects the importance of metacognitive awareness in operational settings. These confidence judgements have direct implications for higher-level decisions concerning the assets that will be deployed to confirm or neutralize the threat—decisions that can have significant operational and safety consequences. The accuracy of these metacognitive monitoring processes (i.e., how well confidence tracks actual performance) is essential because it guides subsequent control processes that regulate both search behavior and decision-making (Metcalfe & Shimamura, 1994). For instance, accurate monitoring of uncertainty during visual search has been shown to guide more effective allocation of attention and adaptive search strategies (Boot et al., 2004; Cunningham & Wolfe, 2014). Similarly, the ability to accurately monitor and assess confidence in perceptual decisions is crucial for optimal detection performance, as it allows appropriate weighting of the degree of certainty when making critical classification decisions (Maniscalco & Lau, 2012; Rahnev et al., 2015).

In the context of ATC-assisted UTD, these metacognitive processes are complicated by the system’s limited transparency. Simple visual cues are not accompanied by information regarding how or why the ATC provided a visual cue. This lack of transparency can contribute to greater uncertainty regarding how operators should interpret the presence of an ATC cue. Consequently, operators must rely on their experience while using the ATC to determine its accuracy and reliability, which in turn shapes their trust and use of the system.

### Fatigue and Automation

The relationship between fatigue and automation use presents unique challenges for understanding operator performance in real-world settings. Characterizing how the interactive factors between human operators and automation assistive technologies influence use and performance can be complex even in convenient, comfortable, and controlled laboratory settings. The military context presents another layer of complexity by introducing operationally relevant factors that can have a profound impact on the nature of human–automation interactions. UTD is a mission-critical task that needs to be performed in a timely matter, any time of day, to ensure safe passage when operating in hostile environments. It is important to note that to perform these missions, operators must first conduct ocean bottom data acquisition missions, which entails deploying and monitoring autonomous vehicles while on the water, before they begin visual inspection of the data to identify and classify threats. Thus, operators can experience high levels of fatigue due to increased time awake while performing UTD, which poses unique issues to visual search performance, let alone use of the automation assistance. A major challenge of performing prolonged visual search is maintaining the proper level of vigilance, which can be impacted by fatigue and sleep loss (Angus & Heslegrave, 1985; Baranski, 2007; Harrision & Horne, 2000; Pilcher & Huffcutt, 1996). Sleep loss heightens the experience of boredom and monotony leading to task disengagement and performance degradation (Gillberg & Akerstedt, 1998; Kjellberg, 1977). For this reason, it is no surprise that the Psychomotor Vigilance Task has been widely used to index sleep deprivation performance decrements (Dinges & Powell, 1985). There is some evidence suggesting motivating and engaging complex tasks are less sensitive to sleep deprivation since motivation, engagement, and expected reward effectively increase arousal (Wilkinson, 1992). Though the high-stakes nature of UTD should increase implicit motivation to perform, the task itself is very monotonous and highly susceptible to vigilance degradation. Moreover, the length of visual search incurs increased burden on operators to maintain vigilance, further compounding task difficulty (Lim and Dinges, 2008). Fatigue due to increased time awake has also been shown to impact metacognitive judgments, specifically judgments concerning confidence in decision-making (Aidman et al., 2019; Baranski, 2007). Metacognitive judgements involve higher-order cognitive processes that are mediated by regions of the prefrontal cortex (PFC), which tend to be more sensitive to sleep deprivation (Goel et al., 2009; Harrison & Horne, 2000). Since confidence judgments directly determine which assets are deployed to confirm or neutralize potential threats, it is critical to understand how these consequential metacognitive abilities are affected by increased time awake.

Results on how fatigue impacts human–automation interactions are nuanced (Finomore et al., 2009; Neubauer et al., 2012). Automation has been shown to reduce information processing load, improving performance under fatigued conditions (Warm et al., 2008). However, the relationship between automation behavior and operator performance is complex. Chavaillez et al. (2020) found that in a luggage screening task, participants detected threats more accurately when the ATC system false alarmed on objects that were noticeably different from the target threat objects, suggesting that certain types of automation errors may actually help maintain operator engagement. However, separate research has shown that overreliance and over-trust in the automation can lead to negative consequences, particularly when operators become complacent about the system’s capabilities (Parasuraman & Riley, 1997). This complacency is especially problematic during sustained attention tasks, where prolonged automation use can degrade situational awareness (Breton & Bossé, 2002) and lead to higher miss and false alarm rates.

### Present Investigation

This present study examined how ATC influences operator UTD performance under conditions of extended wakefulness. UTD is a complex visual search task that is often performed under challenging conditions including heightened fatigue due to increased time awake. While ATC assistance may help operators maintain high performance despite these challenges, it is unclear how fatigue might complicate or diminish the effectiveness of this assistance. To address this, we investigated the potential benefits of ATC integration on target detection performance, as well as metacognitive judgements (i.e., confidence), across multiple sessions over a 24+ hour bout of time awake. A simulated version of a UTD task (simUTD) was created to mirror the real-world military operational task and interface. The ATC also mirrored real-world automation capabilities with imperfect behavior (i.e., the ATC generated false alarm calls and missed true targets). Participants completed four sessions of simUTD over the 24+-hour wakeful period, once without ATC during a high alertness session, and three times with ATC under increased time awake conditions. Critically, the ATC call behavior did not change across sessions to ensure that effects could be attributed to time awake and not changes in the ATC reliability and accuracy. We hypothesized that conducting the simUTD task with ATC would yield better detection and classification performance as indexed by detection sensitivity relative to not having the ATC. Though the research is nuanced concerning the general benefits of ATC on target detection, we believe that the use of an imperfect, yet good ATC system should keep participants from overreliance (i.e., misuse) as well as underutilization (i.e., disuse) of the ATC. We further hypothesized that with ATC, detection and classification performance would not degrade as a function of increased fatigue due to increased time awake. That is, the automation should provide some protection against known performance decrements associated with increased time awake. In terms of the impact of the ATC inclusion on metacognitive judgments as a function of fatigue, we had no clear hypothesis. There is evidence that confidence can inflate with increased fatigue (Bard et al., 1996; Harrison & Horne, 2000), though little is known concerning how ATC cues influence detection confidence judgements. Given this limited knowledge, we embraced an exploratory approach to investigating the relationship between ATC inclusion, metacognitive judgements, and fatigue. Finally, we explored how participant trust in the ATC changed as a function of fatigue and use. Here, we also did not have an a priori hypothesis concerning how fatigue or repeated use would impact overall trust. Although it was possible since use of automation that repeatedly false alarms increases distrust, repeated use with an imperfect ATC across sessions with a liberal detection criterion may decrease trust (Madhavan et al., 2006; Parasuraman, 1997).

## Methods and materials

### Participants

Forty-one active-duty military personnel (*M* age = 29.41 yrs, *SD = *7.83; two female) participated in this study. Data from five participants were excluded because we could not confirm whether they remained awake for the entire 24+-hour wakeful period, which was essential to maintaining the integrity of the fatigue manipulation. Thus, data from thirty-six participants were ultimately analyzed. All procedures were reviewed and approved by the Naval Health Research Center Institutional Review Board (NHRC.2019.006). Participants were recruited from training units and obtained approval to participate from their officer in charge before consenting to the study. All participants were screened for receiving formal training to conduct the UTD task in a military setting as a part of their job. Participants could withdraw their consent at any time without penalty. Participants were compensated $525 USD upon completion of all aspects of the study.

## Task and stimulus

### Simulated Undersea Threat Detection Task (simUTD)

**Fig. 1:**
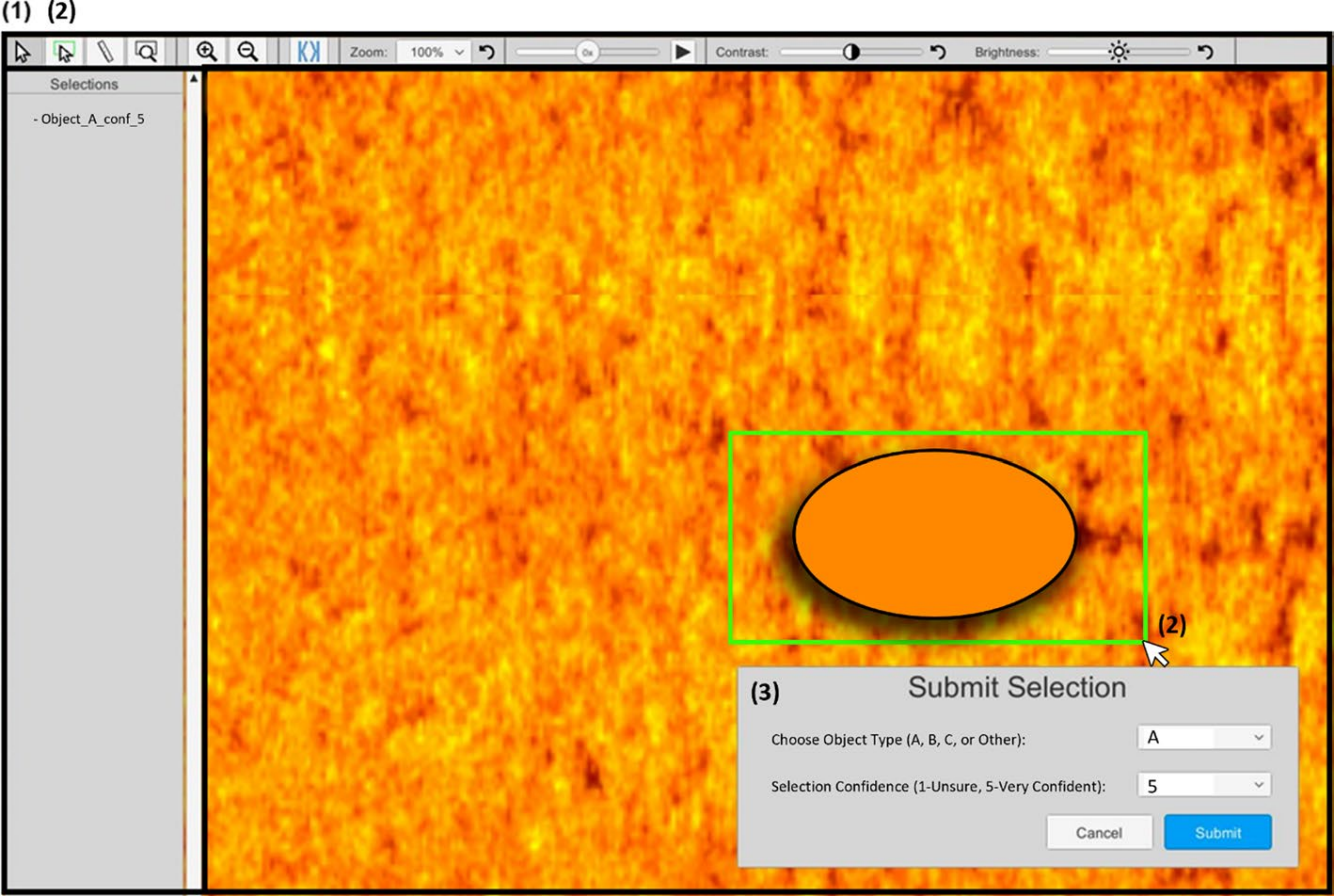
Screenshot of simUTD Task Interface with Target Selection Ssteps *Note.* The cursor (1) in the task toolbar is selected for general navigation of the interface. To highlight an area that contains a potential target object, the selection tool is selected which allows the cursor to be used to click and drag a selection box around the object (2). Then, a dialog box appears with drop-down menus to report “Object Type” and “Selection Confidence” (3). Note: The toolbar at the top of the program contains (from left to right) the cursor, selection tool, ruler tool, magnifier tool, full-screen zoom-in, full-screen zoom-out, re-center screen tool, zoom gradient, waterfall speed adjustment, contrast gradient, and brightness gradient. The orange oval is a placeholder for the target images since the actual target images used in this task cannot be shared for security reasons

The simUTD task was designed using Unity® Development Engine (Unity Technologies, 2017) and developed with input from UTD subject matter experts to accurately simulate the military task. Sonar ocean bottom data collected from practice missions were used as the data sets. Target objects included were rendered “target-like objects” that matched real undersea target types and were synthetically embedded in the ocean bottom data sets. For security purposes, exact descriptions and depictions of the target object types cannot be shared. The objective of the simUTD task was to search through sonar scans of ocean bottom for objects matching one of three possible target object types (A, B, or C) (see Fig. 1 for simUTD task example). Before each simUTD session, participants were instructed to search for a primary target object type. This search instruction mirrors actual UTD missions where operators are directed to find all objects that match the “order of battle” target type. In addition to the primary target type, participants also searched for objects that fit the other two target types. Although, as with actual UTD, finding objects that matched the primary target type was the most critical to task performance since hit and false alarm rates were determined based on (in) correct detection and misses of the primary target objects in the simUTD session.

The ocean bottom data were presented from a birds-eye perspective looking down at the ocean floor (See Fig. 1). Participants vertically scrolled through the data manually with a mouse scroll wheel or automatically by toggling the speed-adjustable waterfall feature at the top bar menu of the screen. The pixel color/brightness scale varied from black to bright orange, and the contrast and brightness could be controlled using the labeled sliders in the top menu bar. Upon seeing a target object during search, participants used their mouse to click and drag a square around the location of the object to highlight its location on the screen. A dialog box appeared with a drop-down menu to select the 1) Object Type (A, B, C, or Other) and 2) Confidence in Detection Rating (1–5 scale, with 1 indicating little to no confidence and 5 indicating maximal confidence). Once both drop-down selections were made, the detection call information appeared on the left side of the screen in the “Selections” list. As more detection calls were made, they were added to the “Selections” list. Clicking on a previously made call listed in the “Selections” list took the participant back to the location of the call in the data set, allowing them to edit or update previously made call selections. Total progress was also saved, and participants could click “Checkpoint” in the call list to return to the current progress location. The menu bar also included zoom and measurement tools. Like the selection tool, the zoom tool allowed participants to click and drag around an area to zoom-in on and the measurement tool allowed participants to click and drag to display the estimated measurement from the click to where the mouse is dragged in meters.

For ATC-included sessions, the ATC appears as a dashed circle around the location of an object that potentially matched the primary target type. Prior to each session, participants were told that the ATC was trained to cue the primary target type only and should therefore only cue objects that matched the primary target object type. When the dashed circle was clicked on, the ATC adjudication dialog box with “Accept” and “Reject” buttons appeared in the center of the screen. If the “Accept” button was clicked, the dashed circle turned green, indicating agreement that the ATC had accurately cued an object that matched the primary target type. However, if the “Reject” button was clicked, the dashed circle turned red, indicating the ATC had false alarmed. Following acceptance or rejection of the ATC call, participants proceeded with highlighting the object and selecting object type and confidence rating. Like the detection calls, ATC adjudications could be edited and updated.

For security reasons, specific parameters of the ATC system's performance (i.e., hit and false alarm rate) cannot be disclosed. The system was designed to mirror fielded capabilities, exhibiting both successful target detections and false alarms at rates consistent with currently deployed systems. The ATC's behavior remained constant across all sessions to ensure that any observed changes in performance could be attributed to increased time awake rather than variations in ATC reliability.

### Simulated Ocean Bottom Data Sets

Six continuous scan ocean bottom sonar images were generated as the stimulus set for this study. Each ocean bottom data set was roughly equated in terms of length (measured in square nautical miles), ocean bottom type complexity (i.e., smooth sand, sand ripple, rocks, and rock formations), and number of man-made objects (i.e., fish traps, sunken ships, debris, etc.). Each ocean bottom data set contained five objects that fit the primary target type and five objects that fit the other target types, totaling ten target objects to detect and classify. For counterbalancing purposes, each simulated ocean bottom data set was generated for a specific primary target type condition. ATC-included datasets were the same as datasets without ATC; however, ATC datasets contain ATC calls (indicated by dashed circles). ATC datasets were equated in terms of number of ATC false alarm calls and ATC target object type hit calls per square nautical mile.

## Measures

### Fatigue Science Readiband (Alertness score)

To measure participant fatigue level and ensure that participants did not sleep in the interim between Visits 2 and 3, we issued Fatigue Science Readibands (Vancouver, Canada) to participants to wear for the duration of the study. The Readiband is a wrist wearable containing an accelerometer that continuously collects user’s actigraphy. These data are passed to the Sleep Activity and Task Effectiveness (SAFTE) model (Hursh et al., 2004), which simulates the physiological determinants of fatigue based on processes that include (1) a sleep reservoir, (2) hours of wakefulness, (3) current sleep debt, (4) the circadian timing of sleep and sleep fragmentation, (4) sleep inertia process, and (5) light exposure based on geographic location (Russell et al., 2000). In this study, alertness scores produced by the SAFTE Model were used as a physiological measure of fatigue. Alertness scores ranged from 0 to 100, with scores below 70 indicating high susceptibility to human factors risks due to fatigue. Previous research has independently validated the SAFTE model against subjective ratings of fatigue and cognitive performance (Hursh et al., 2006), as well as shown it is predictive of human factors accident risks (Van Dongen, 2004).

### Karolinska Sleepiness Scale

The Karolinska Sleepiness Scale (KSS; Åkerstedt & Gillberg, 1990) is a nine-point anchored scale with the following steps: 1 (extremely alert), 2 (very alert), 3 (alert), 4 (fairly alert), 5 (neither alert nor sleepy), 6 (some signs of sleepiness), 7 (sleepy, but no effort to keep alert), 8 (sleepy, some effort to keep alert), and 9 (very sleepy, great effort to keep alert, fighting sleep). The KSS has been used extensively in the sleep and fatigue literature and validated against electroencephalography and performance metrics (Kaida et al., 2006).

### Signal Detection Variables

Hits, misses, false alarms, and correct rejections were used to calculate signal detection variables as well as metacognitive variables. For scoring purposes, a hit was defined as correctly identifying a primary target object and, in ATC-included sessions, accepting a valid ATC call. A false alarm occurred when participants either incorrectly identified a non-target or other target object as a primary target and/or accepted an invalid ATC call. A correct rejection was recorded when participants correctly identified other target objects and, during ATC-included sessions, appropriately rejected invalid ATC calls. Hit rate was the number of hits divided by the sum of hits and misses ($\frac{\text{Hits}}{\text{Hits}+\text{Misses}}$). False alarm rate was the number of false alarms divided by the sum of false alarms and correct rejections ($\frac{\text{False Alarms}}{\text{False Alarms}+\text{Correct Rejections}}$). Because target prevalence was low making it difficult to determine equal variances between *signal*+*noise* and *noise* distributions, the nonparametric *A’* was used as the measure of detection sensitivity (Donaldson, 1993). *A’* is an estimate of the area under the receiver operator characteristic (ROC) curve based on individual probability of hit and probability of false alarm rates. *A’* values therefore range from 0 to 1, with 0 reflecting maximally poor sensitivity and 1 reflecting maximal sensitivity. *B”* was used as the measure of detection bias and is monotonically related to the slope of the ROC curve for a pair on an individual’s probability of hit and false alarm rates. *B”* slope values less than zero indicate the magnitude of bias toward a liberal detection criterion, values greater than zero indicate the magnitude of bias toward a conservative detection criterion, and values of zero indicate an unbiased detection criterion.

**Fig. 2:**
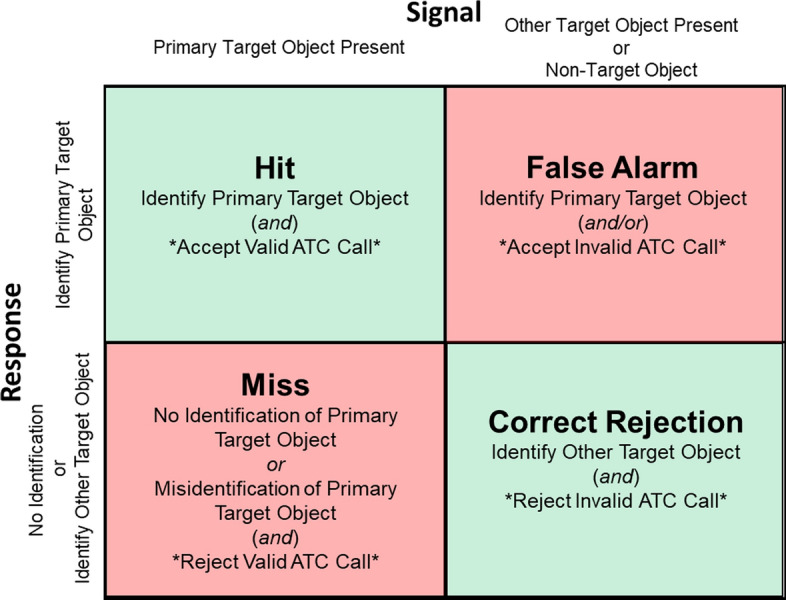
Signal Detection Ttable. *Note* 2x2 signal detection table with hit, miss, false alarm, and correct rejection criterion. Asterisks (*) highlight additional criterion required when performing simUTD task with ATC

Metacognitive sensitivity was calculated as the area under the type 2 receiver operating characteristic (AUROC2) curve (Fleming & Lau, 2014). For each detection decision, participants provided a confidence rating on a 1–5 scale, where 1 indicated little or no confidence and 5 indicated maximal confidence in their detection and classification decision. These confidence ratings, paired with the accuracy of each detection (hit vs. false alarm), were used to compute metacognitive sensitivity. AUROC2 assesses how well participant’s confidence judgements discriminated between their correct and incorrect detection decisions, with higher values indicating better metacognitive discrimination. For example, high metacognitive sensitivity would be reflected in consistently higher confidence ratings for hits compared to false alarms, while poor metacognitive sensitivity would be indicated by similar confidence ratings regardless of decision accuracy (Fig. 2).

### Automatic Target Cuing Disuse and Misuse

Parasuraman (1997) defined key use behaviors that arise when humans interact with automation, which include misuse and disuse. Misuse is the overreliance on, in this case, the ATC when its cues are invalid. Since the ATC was programmed to be imperfect to mirror fielded capability, it cued non-target objects (i.e., false alarm). Acceptance of these false alarm calls would lead to inflated overall false alarm rates. We therefore quantified misuse as $(\frac{\text{Accepted Incorrect ATC Calls}}{\text{Total Seen Incorrect ATC Calls}})$, with 0% indicating calibrated use of the ATC (i.e., rejecting all ATC false alarms) and 100% indicating maximal overreliance on the ATC (i.e., accepting all ATC false alarms).

Disuse is the underutilization of the ATC when its cues are valid. The ATC was programmed to be good, cuing a high proportion of the primary target objects in the data set. Rejecting these correct ATC cues would lead to a low overall hit rate and thus would result in poor task performance. We therefore quantified disuse as $100\%- (\frac{\text{Accepted Correct ATC Calls}}{\text{Total Seen Correct ATC Calls}})$, with 0% indicating calibrated use of the ATC (i.e., accepting all ATC hits) and 100% indicating total underutilization of the ATC (i.e., rejecting all ATC hits). $(\frac{\text{Accepted Correct ATC Calls}}{\text{Total Seen Correct ATC Calls}})$ was subtracted from 100% in order to make it easier to visually compare misuse and disuse. That is, calibrated use is 0%, and values greater than 0% on both scales indicate the magnitude of misuse and disuse.

### Trust in Automatic Target Cuing Checklist

We adapted the Checklist for Trust between People and Automation (Jian et al., 2000) to the context of interaction with ATC, by replacing “automation” with “Automatic Target Cuing System” for each item when relevant (See Supplemental Materials). This adapted “Trust in ATC Checklist” was administered after each ATC-included simUTD session to assess trust in the ATC. Like the original checklist, the Trust in ATC checklist is a self-report measure that includes 12 items that sample factors of trust (i.e., confidence, integrity, reliability, and familiarity) and distrust (i.e., perceived deceptiveness, suspiciousness, likelihood of injurious outcomes). Participants rated the degree of agreement, or disagreement, with each item using a 1–7 Likert scale, with 1 indicating “Not at all” and 7 indicating “extremely.” Based on experiments validating the measure (Jian et al., 2000), trust and distrust can be conceptualized as opposite extremes along a single dimension.

### Design and Procedure

A 2 (automatic target cuing: included, not included) x 4 (time of task: 0700, 1800, 0030, 0700-next day) within-subject experimental design was used, with the levels of ATC being counterbalanced such that each participant attended one simUTD session without ATC at either 0700 or 1800. Participants experienced ATC-included simUTD task for all other sessions (i.e., 0030 and 0700 next day). Specifically, odd participants performed the simUTD task without ATC for session 1 (0700) and the simUTD task with ATC included for sessions 2 (1800), 3 (0030), and 4 (0700-next day). Even participants performed the simUTD task without ATC for session 2 (1800) and the simUTD task with ATC included for sessions 1 (0700), 3 (0030), and 4 (0700-next day). This counterbalancing procedure was chosen to ensure that each participant performed the simUTD task once without ATC during a time when they should be at high levels of alertness according to normal circadian rhythms and the simUTD task with ATC included for sessions where participants were expected to experience greater fatigue due to sleep loss. Research on healthy adults with normal circadian rhythms supports the notion that 0700 and 1800 are effectively similar in terms of how time awake affects general alertness, vigilance, and attention control performance (Duffy et al., 2009; Goel et al., 2013; Killgore, 2010). Furthermore, our previous work demonstrated no differences in simUTD task performance, perceived fatigue, or fatigue level as determined by an actigraphy-based wearable between 0700 and 1800 sessions (Dunn et al., 2023). Therefore, this counterbalancing procedure ensured one control session (ATC not included during 0700 or 1800) for each participant, multiple sessions of ATC included at intervals of increasingly greater experienced fatigue, while limiting the study to one ~24-hour wakeful period to adhere to participant recruitment constraints.

Visit 1 occurred three days prior to a participant’s laboratory visits (i.e., Visits 2 and 3). During Visit 1, participants were provided with informed consent, outfitted with a Readiband to be worn while enrolled in the study, and given instructions in preparation for the in-laboratory visits comprising the ~24-hour wakeful period (i.e., Visits 2 and 3). Critically, participants were informed that their data would be unusable if they used caffeine, nicotine, or alcohol at any point during the ~24-hour wakeful period. If participants identified as caffeine or nicotine users, they were encouraged to minimize their consumption in anticipation of the ~24-hour wakeful period.

Participants arrived for Visit 2 at 0630 which marked the start of the ~24-hour wakeful period. During Visit 2, participants completed one simUTD session. All simUTD sessions were performed in a soundproof booth (WhisperRoom Inc.) equipped with an adjustable desk, chair, high-resolution monitor, standard keyboard, and mouse. A webcam mounted to the top of the booth monitor was used by the experimenters to verify participants stayed awake for the duration of the session. Prior to each simUTD session, the experimenter read through the task instructions, which indicated the session’s primary target object (see Supplementary Materials for Task instruction templates). For ATC-included simUTD sessions, participants were further instructed that the ATC system was tuned to the primary target object type for that session. Participants were allotted 180 minutes maximum to complete each simUTD session. Before and after each simUTD session, participants reported their perceived level of alertness/sleepiness using the KSS. After each ATC-included simUTD session, participants completed the Trust in ATC Checklist. Once participants completed simUTD 1, they were allowed to leave the laboratory to return to their normal daily activities. Participants were instructed to refrain from naps, nicotine, caffeine, and alcohol between Visits 2 and 3 and reminded to continue to wear the issued Readiband to ensure no naps were taken between Visits 2 and 3.

**Fig. 3:**
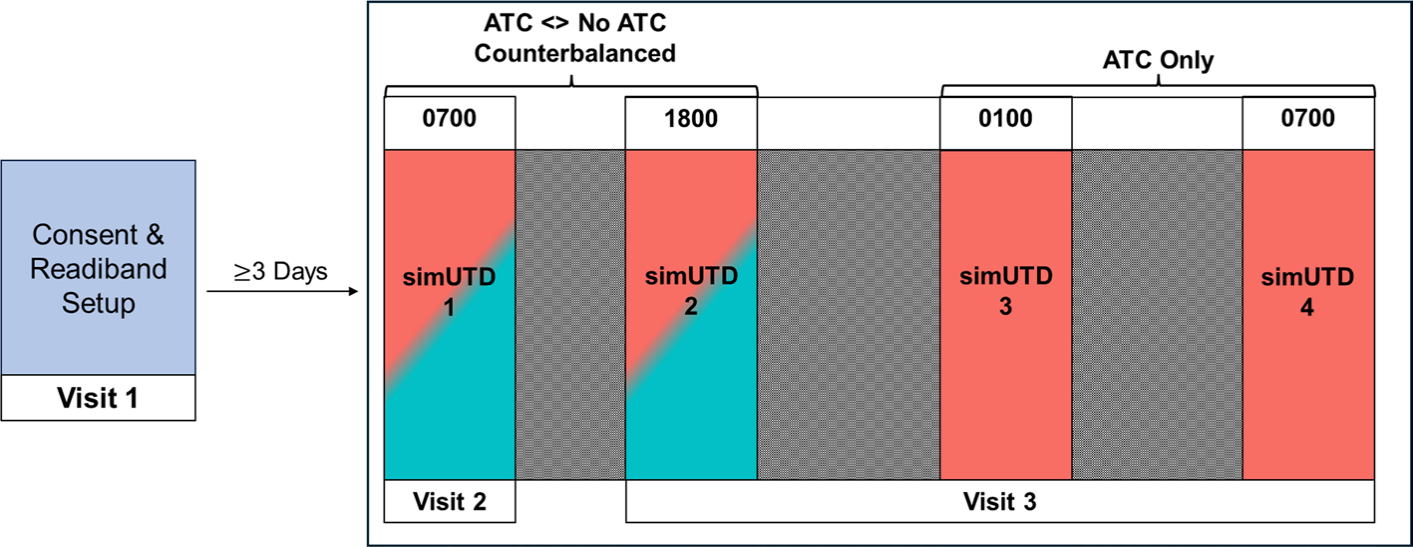
Schematic of Experimental Protocol Flow. *Note* Visit 1 was conducted at least 3 days prior to Visits 2–3. Four simulated undersea threat detection (SimUTD) task sessions were performed over the ~ 24-hour time awake period. Blue–red shading for simUTD 1 & 2 depicts counterbalancing method entailing each participant performed simUTD without ATC once either at 0700 or 1800. All participants performed ATC-included simUTD for simUTD 3 & 4

Participants returned to the laboratory for Visit 3 at 1730. Upon arrival, participants were given a breathalyzer to confirm no alcohol had been consumed during the break. At this time, female participants were given a pregnancy test to verify fitness for continuation with the sleep restriction portion of the study. During Visit 3, participants remained in the laboratory to perform three more simUTD sessions (see Figure 3 for timing of simUTD sessions). Participants were allowed to engage in leisurely activities (e.g., watching television, video games, reading, etc.) between simUTD sessions under the watch of researchers. After simUTD 4 was completed, participants were compensated, and experimenters ensured participants were provided a ride home by an alert individual who did not take part in the study.

### Analysis Approach

All analyses were conducted in R (R Core Team, 2019). All visuals were created using *ggplot2* (Wickham, 2016). A strict criterion was employed to calculate hit and misses. That is, a hit was the correct detection and identification of primary target objects. Additionally, for ATC-included sessions, correct ATC calls on primary target objects also had to be “accepted” in order for the participant call to be considered a hit. Conversely, absence of a detection call, misidentification, or rejection of a correct ATC call for primary target objects was considered misses. False alarms, on the other hand, were labeled based on a more liberal criterion factored other target objects in addition to the primary target object into the assessment. For example, labeling another target object as a primary target object was a false alarm, as was labeling debris or ocean bottom features as a primary or other target object. Furthermore, accepting an incorrect ATC call would qualify as a false alarm. Correct rejections were also determined in a similar manner to hits. A correct rejection was the correct detection and identification of other target objects and additionally a “rejection” of the incorrect ATC call when relevant.

For inference analysis, we used a Bayesian hypothesis testing approach. Bayes factors (BFs) were computed using the *BayesFactor* package (Morey & Rouder, 2014). A default prior of *r *= 2/2 (i.e., “Medium”) was utilized for analyzing differences in means, and a default fixed r scale = ½ was utilized for ANOVAs and t-tests. For ANOVA results, we used a model comparison procedure where any factor(s) demonstrating a BF > 3 against the error-only random effect (i.e., value ~ participant) were compared against each other (e.g., a main effect model vs. interaction model). Evidence categories for BFs follow Lee and Wagenmakers (2013): 1–3 “anecdotal/weak,” 3–10 “moderate,” 10–30 “strong,” 30–100 “very strong,” > 100 “extreme.” All tests are non-directional and are presented in terms of the alternative hypothesis (BF_ALT_; some difference) or null (BF_NULL_; no difference) when a given BF > 1. Post hoc tests were not performed for results showing “anecdotal/weak” evidence in favor of the alternative hypothesis. For each BF result, Monte Carlo Markov chain (MCMC) samples were extracted from the posterior distribution of the effect size and used to compute the 95% highest density interval (HDI) as the effect size credible interval range and the mean standardized effect size (*δ*) as the descriptive estimate of the effect size (Kruschke, 2014). The *BayesTestR* package was used to calculate the posterior distribution of the effect size and extract MCMC samples (Makowski et al., 2019). Magnitude of the mean standardized effect size (*δ*) can be interpreted similar to Cohen’s *d,* with effect sizes of *δ* = 0.2 being small, *δ* = 0.5 being medium, and δ $\ge \hspace{0.17em}$0.8 being large. Moreover, interpreting δ with the 95% HDI further indicates the degree of certainty that should be allocated to the effect size estimate.

In addition to analyses conducted to test the stated hypotheses, analyses assessing order effects produced by counterbalancing (i.e., when the baseline No ATC session was completed) were also performed. Specifically, between-subjects Bayesian t-tests were conducted to identify differences between and within ATC inclusion conditions (i.e., No ATC vs. ATC) at baseline (i.e., session 1–0700 vs. session 2–1800). For all analyses testing, the stated hypotheses measures from sessions 1 and 2 were combined into a single baseline measure.

Common biomathematical models of alertness over a 24-hour period are generally based on the Borbély two-process model, which comprised of a homeostatic and circadian processes that interact to determine level of arousal and sleep pressure as a function of time awake (Borbély et al., 2016; Mallis et al., 2004). The homeostatic process is modeled by a logarithmic function, whereas the circadian process is modeled approximately by a single-cycle sinusoidal function that tracks daylight. Alertness during normal waking daylight hours remains relatively stable, though with sensitive measures slight fluctuations such as the “circadian trough” can be observed (Killgore, 2010). Thus, linear mixed-effects models (LMMs) were generated up to the fourth-order polynomial (quartic) to determine the pattern of alertness over ~24-hour time awake cycle since, for sensitive measures, general fluctuations driven by the homeostatic and circadian processes as well as the characteristic dip and recovery in the afternoon should be best fit by the quartic model. Linear mixed-effect models (LMM) were fit using the *lme4* package (Bates et al., 2015), and *F*-value corrections use the Satterthwaite’s method for LMM. Pairwise LMM model comparisons were conducted between the quartic model and each of the simpler models (i.e., linear, quadratic, and cubic). These comparisons were performed by using likelihood ratio tests which yielded Chi-squared (*χ*^2^) and *p* values to determine the statistical significance of the differences in fit. Akaike Information Criterion (AIC) values were also calculated and compared across models.

The original version of the Trust in Automation Checklist published by Jian and colleagues’ (2000) was designed to produce an overall trust in automation score by reverse scoring distrust items and summing all response values. Greater overall scores therefore indicate greater trust in the automation. Though trust and distrust can be conceptualized as opposite extremes along a single dimension, in developing the Trust in Automation checklist Jian and colleagues’ (2000) also found that trust and distrust can be also conceptualized as separable constructs that could be treated differently. We performed principal components analysis (PCA) using Varimax rotation on the Trust in ATC Checklist items across sessions using the *psych* package. In performing this analysis, we were not only interested in replicating the factor loadings of all trust and all distrust items on separable trust and distrust components but also whether these factor loadings were stable as a function of fatigue. To accomplish this, only components with eigenvalues greater than 1 were retained (Kaiser Criterion; see Kaiser, 1960 for description). Item loadings for components greater than 1 were extracted and evaluated to determine correspondence to factor analysis results reported by Jian and colleagues’ 2000.

## Results

### Fatigue Monitoring

#### Readiband (Alertness Scores)

**Fig. 4:**
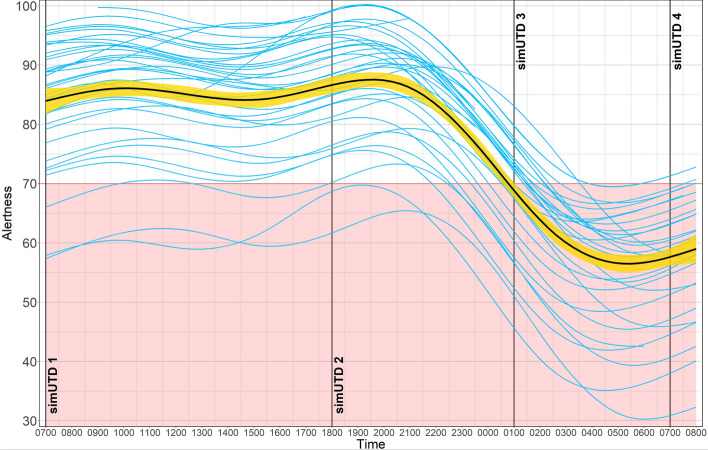
Individual and Group Average Readiband Alertness Scores during ~24-hour Wakeful Period. *Note* Individual blue lines represent individual operator Alertness scores determined by the SAFTE model). The bold black line represents the nonlinear function from a generalized additive model computed for the entire sample, and the yellow band represents the standard error. Vertical lines denote the start of each simUTD session. Individuals falling below 70 (red block) are in a state of fatigue impairment based on the SAFTE model

Figure 4 shows the known circadian-based nonlinear pattern of alertness scores derived from the SAFTE model for all participants. We found that LMM model comparison using likelihood ratio tests and AIC favored the quartic model (AIC = 11658.90) over the linear (AIC = 15335.22, *χ*^2^ (15, *N* = 36) = 3442.2, *p* < 0.0001), quadratic (AIC = 13106.69, *χ*^2^ (11, *N* = 36) = 1279.1, *p* < 0.0001), and cubic (AIC = 12836.58, *χ*^2^ (6, N = 36) = 1009.2, *p* < 0.0001) models.

Pairwise within-subject Bayesian t-tests on alertness scores prior to each simUTD session revealed that alertness was higher prior to session 2 (1800; *M* = 86.80, *SD = *9.90) relative to session 1 (0700; *M = *83.17, *SD = *10.23, BF_ALT_ = 6229.46, *δ* = −1.01, 95% HDI [− 1.48, − 0.54]). Alertness was higher prior to session 1 than session 3 (0100; *M = *69.17, *SD = *9.47, BF_ALT_ > 100,000, *δ* = 2.23, 95% HDI [1.55, 2.94]) and session 4 (0700 next morning; *M = *58.12, *SD = *10.69, BF_ALT_ > 100,000, *δ* = 7.00, 95% HDI [5.06, 8.90]). Similarly, alertness was higher prior to session 2 than session 3 (BF_ALT_ > 100,000, *δ* = 2.97, 95% HDI [2.14, 3.87]) and session 4 (BF_ALT_ = >100,000, *δ* = 30.32, 95% HDI [22.45, 38.61]). Finally, alertness was higher prior to sessions 3 than session 4 (BF_ALT_ > 100,000, *δ* = 1.72, 95% HDI [1.12, 2.30]). Thus, overall alertness was highest prior to session 2, followed by session 1, session 3, and lowest prior to session 4.

#### Subjective Sleepiness (Karolinska Sleepiness Scale)

**Fig. 5:**
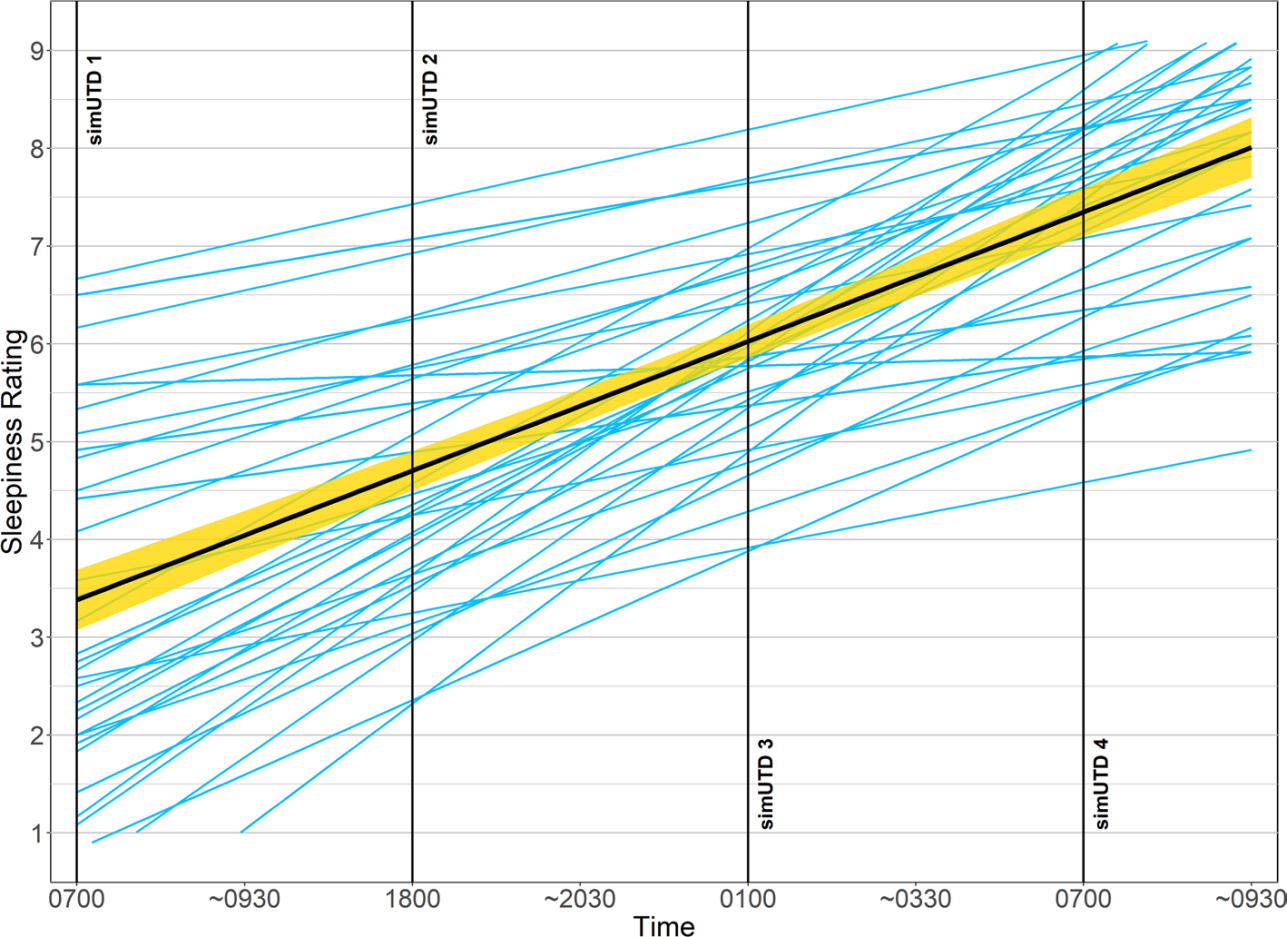
Subjective Sleepiness Ratings (Karolinska Sleepiness Scale). *Note* Individual blue lines represent individual participant KSS ratings. The bold black line represents the linear function computed for the entire sample, and the yellow band represents the standard error. Vertical lines denote the start of each simUTD session

A clear linear increase in subjective sleepiness ratings as a function of time (*b* = 0.69, SE = 0.039, t = 17.50, *p* < 0.0001; see Figure 5)*.* Post-simUTD KSS ratings were the same as pre-simUTD ratings, except for three individual post-simUTD ratings. Thus, time on task did not increase perceived sleepiness. It is worth noting that the three post-simUTD ratings that differed from pre-simUTD ratings differed by $\le$ 2 points on the KSS scale. Furthermore, the three post-simUTD ratings that differed from pre-simUTD ratings were from different participants and occurred at the end of different sessions (i.e., sessions 2, 3, and 4). Consequently, there were no noteworthy patterns to account for the three instances of minimal time-on-task effects. As such, for subsequent analyses we only used pre-simUTD KSS ratings since analysis of post-simUTD KSS ratings would yield highly similar results.

Pairwise within-subjects Bayesian t-tests were conducted to identify differences in KSS ratings as a function of session. Participants reported similar levels of sleepiness prior to sessions 1 (*M = *4.00, *SD = *1.48) and 2 (*M = *4.13, *SD = *1.02, BF_NULL_ = 5.02, *δ* = − 0.075, 95% HDI [−0.39, 0.23]). Reported sleepiness was greater prior to sessions 3 (*M = *6.44, *SD = *0.94, BF_ALT_ > 100,000, *δ* = − 1.25, 95% HDI [− 1.69, − 0.80]) and 4 (*M = *8.05, *SD = *1.24, BF_ALT_ > 100,000, δ = − 1.88, 95% HDI [− 2.48, − 1.35]) relative to session 1. Participants reported being less sleepy prior to session 2 than session 3 (BF_ALT_ > 100,000, δ = − 1.73, 95% HDI [− 2.29, − 1.22]) and session 4 (BF_ALT_ = >100,000, *δ* = − 2.28, 95% HDI [− 2.93, − 1.67]). Reported sleepiness was greater prior to session 4 than sessions 3 (BF_ALT_ > 100,000, *δ* = − 1.15, 95% HDI [− 1.57, − 0.72]). Thus, overall sleepiness was lowest prior to sessions 1 and 2, followed by session 3, and highest prior to session 4.

### simUTD Task Performance

#### Total Task Time

To evaluate potential order effects produced by the baseline counterbalancing method, between-subjects Bayesian t-tests were conducted to identify differences between and within ATC inclusion conditions (i.e., No ATC vs. ATC) at baseline (i.e., sessions 1 vs. 2). Participants were slower to complete simUTD without ATC during session 1 (*M = *104.55 mins, *SD = *25.29 mins) compared to session 2 (*M = *73.46 mins, *SD = *15.33 mins, BF_ALT_ = 10.94, *δ* = 0.89, 95% HDI [0.22, 1.59]). However, participants completed the task with ATC in a similar amount of time for sessions 1 (*M = *112.15 mins, *SD = *27.40 mins) and 2 (*M = *94.28 mins, *SD = *15.41 mins, BF_NULL_ = 1.53, *δ* = 0.35, 95% HDI [− 0.26, 0.96]). For session 1, there were no differences in total task time between participants who performed simUTD with versus without ATC (BF_NULL_ = 2.73, *δ *= − 0.14, 95% HDI [− 0.75, 0.45]). For session 2, total task time was similar between participants who performed simUTD with versus without ATC (BF_ALT_ = 1.71, *δ* = − 0.56, 95% HDI [− 1.22, 0.05]).

A within-subjects Bayesian t-test was conducted that compared ATC inclusion conditions during the baseline session. Analysis yielded weak evidence in favor of the alternative hypothesis (BF_ALT_ = 1.21, *δ* = − 0.32, 95% HDI [− 0.65, 0.0]) indicating no differences in total task time between ATC inclusion conditions at baseline. SimUTD with ATC was completed on average in 103.21 minutes (*SD = *27.25 mins), and simUTD without ATC was completed on average in 89.01 minutes (*SD = *24.16 mins).

**Fig. 6:**
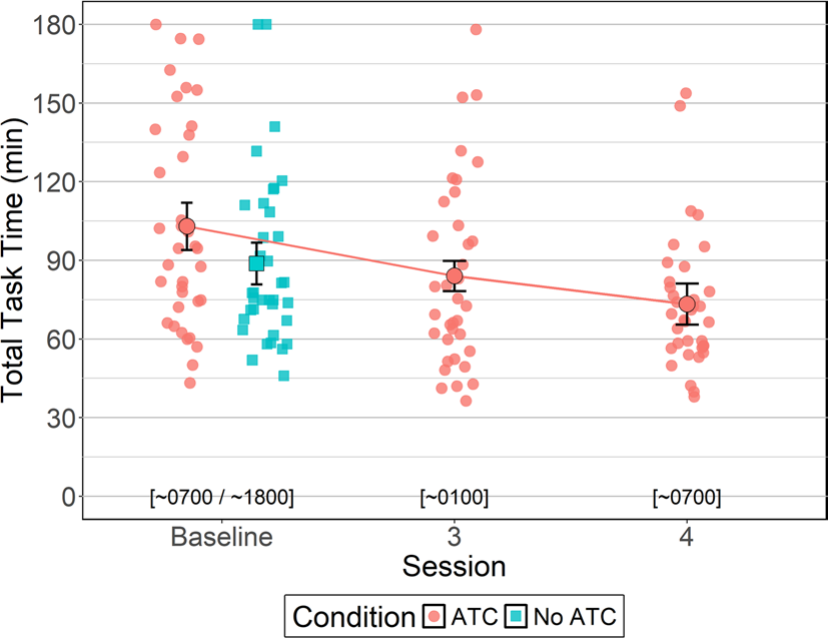
Total Task Time by ATC Condition and Session. *Note* Error bars represent within-subject confidence intervals

A Bayesian analysis of variance (ANOVA) was conducted to evaluate whether total task time changed as a function of fatigue when simUTD was performed with ATC. Bayesian ANOVA yielded extreme evidence in favor of the alternative hypothesis (BF_ALT_ = 3270.88, *δ* = 0.91, 95% HDI [0.04, 2.98]) indicating that total task time differed across sessions. Pairwise within-subjects Bayesian t-tests were conducted between sessions (baseline vs. session 3 vs. session 4) to identify the pattern of change as a function of fatigue. Participants were slower baseline (*M = *103.21 mins, *SD = *27.25 mins) than session 3 (*M = *84.00 mins, *SD = *16.86 mins, BF_ALT_ = 28.14, *δ* = 0.54, 95% HDI [0.19, 0.89]) and session 4 (*M = *73.30 mins, *SD = *23.31 mins, BF_ALT_ = 414.07, *δ* = 0.72, 95% HDI [0.36, 1.10]). However, participants completed the task in a similar amount of time for sessions 3 and session 4 (BF_ALT_ = 1.53, *δ* = 0.33, 95% HDI [0.01, 0.66]) (Fig. 6).

#### Hit Rate

Between-subjects Bayesian t-tests were conducted to identify potential order effects for hit rate (i.e., the proportion of correctly identified primary target objects relative to total primary target objects). For simUTD without ATC, hit rates were similar between sessions 1 (0700; *M = *71.9%, *SD = *31.5%) and 2 (1800; *M = *85.5%, *SD = *16.8%, BF_NULL_ = 1.00, *δ *= − 0.46, 95% HDI [− 1.12, 0.13]). Hit rates were similar between sessions 1 (*M = *81.1%, *SD = *20.4%) and 2 (1800; *M = *77.7%, *SD = *30.1%, BF_NULL_ = 2.98, *δ *= 0.084, 95% HDI [− 0.47, 0.67]) with ATC. Also, hit rates between ATC inclusion conditions did not differ for session 1 (BF_NULL_ = 2.13, δ = − 0.25, 95% HDI [−0.89, 0.30]) or session 2 (BF_ALT_ = 2.29, *δ* = 0.23, 95% HDI [− 0.35, 0.81]).

To test whether ATC inclusion provided a detection performance benefit relative to No ATC at high alertness times, a within-subjects Bayesian t-test was conducted that compared hit rates between ATC inclusion conditions during baseline. Analysis yielded moderate evidence in favor of the null hypothesis (BF_NULL_ = 5.51, *δ* = − 0.024, 95% HDI [− 0.34, 0.29]) indicating hit rates did not differ between ATC inclusion conditions at baseline. Hit rates with ATC were 79.4% on average (*SD = *26.1%) and without ATC were 78.7% on average (*SD = *26.2%).

**Fig. 7:**
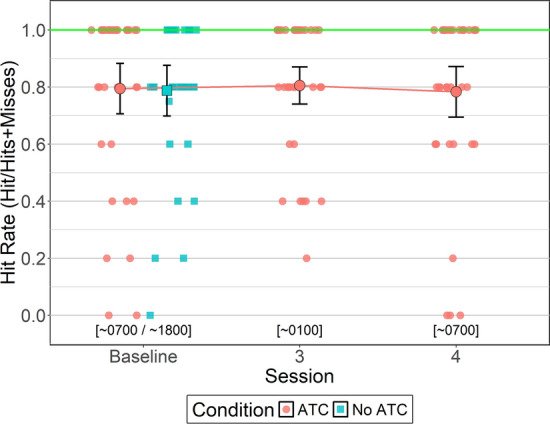
Hit Rate by ATC Condition and Session. *Note* Error bars represent within-subject confidence intervals

A Bayesian ANOVA was conducted to evaluate whether ATC-included simUTD hit rates changed as a function of fatigue. Bayesian ANOVA yielded strong evidence in favor of the null hypothesis (BF_NULL_ = 10.91, δ = 0.32, 95% HDI [0.01, 0.86]) indicating that hit rates did not differ across ATC-included simUTD sessions. Baseline hit rate was 79% (*SD = *2.6%), session 3 hit rate was 80.5% (*SD = *1.9%), and session 4 hit rate was 78.3% (*SD = *2.6%) (Fig. 7).

#### False Alarm Rate

Between-subjects Bayesian t-tests were conducted to identify potential order effects for FA rate (i.e., the proportion of non-target objects incorrectly identified as primary target objects, including incorrect acceptance of invalid ATC calls). For simUTD without ATC, FA rates did not differ between sessions 1 (*M = *79.5%, *SD = *13.1%) and 2 (*M = *80.8%, *SD = *6.8%, BF_NULL_ = 2.95, *δ* = 0.085, 95% HDI [− 0.50, 0.68]). However, for ATC-included simUTD FA rates were higher for session 1 (*M = *87.1%, *SD = *10.5%) than 2 (*M = *77.1%, *SD = *6.7%, BF_ALT_ = 4.28, *δ* = 0.72, 95% HDI [0.05, 0.72]). FA rates were similar between ATC inclusion conditions for both sessions 1 (BF_NULL_ = 2.23, *δ* = − 0.23, 95% HDI [− 0.84, 0.33]) and 2 (BF_ALT_ = 2.38, *δ* = 0.21, 95% HDI [−0.35, 0.78]).

To test whether ATC inclusion reduced FA rates relative to No ATC at high alertness times, a within-subjects Bayesian *t*-test was conducted that compared FA rates between ATC inclusion conditions during baseline. Analysis yielded moderate evidence in favor of the null hypothesis (BF_NULL_ = 5.14, *δ* = − 0.06, 95% HDI [− 0.37, 0.26]) indicating there were no FA rate differences between ATC inclusion conditions at baseline. That is, FA rates for simUTD performed with ATC (*M = *82.1%, *SD = *10.0%) were the similar to simUTD performed without ATC (*M = *80.2%, *SD = *10.5%).

**Fig. 8:**
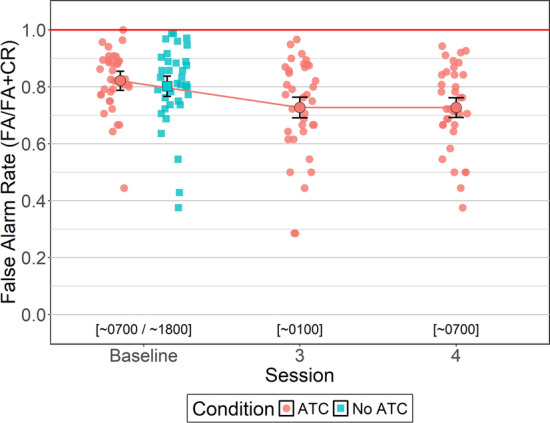
False Alarm Rate by ATC Condition and Cession. *Note* Error bars represent within-subject confidence intervals

A Bayesian ANOVA was conducted to evaluate whether ATC-included FA rates changed as a function of fatigue. Bayesian ANOVA yielded very strong evidence in favor of the alternative hypothesis (BF_ALT_ = 38.5, *δ* = 0.66, 95% HDI [0.02, 1.86]) indicating that FA rates differed across sessions. FA rates were higher at baseline than sessions 3 (*M = *72.7%, *SD = *10.7%, BF_ALT_ = 13.89, *δ *= 0.51, 95% HDI [0.16, 0.84]) and 4 (*M = *72.7%, *SD = *10.1%, BF_ALT_ = 6.52, *δ* = 0.45, 95% HDI [0.12, 0.81]). However, FA rates were similar between sessions 3 and 4 (BF_NULL_ = 5.48, δ = 0.028, 95% HDI [− 0.28, 0.33]) (Fig. 8).

#### Detection Sensitivity (A’)

Between-subjects Bayesian t-tests were conducted to identify potential order effects for detection sensitivity (i.e., *A’*, which measures ability to discriminate between target and non-target objects, with values ranging from 0 to 1 where higher values indicate better discrimination). For No ATC, there were no differences in detection sensitivity between sessions 1 (*M = *0.52, *SD = *0.27) and 2 (*M = *0.59, *SD = *0.17, BF_NULL_ = 2.43, *δ* = − 0.19, 95% HDI [−0.81, 0.35]). Likewise, for ATC-included simUTD detection sensitivity was similar between sessions 1 (*M = *0.57, *SD = *0.22) and 2 (*M = *0.59, *SD = *0.24, BF_NULL_ = 3.08, *δ* = − 0.33, 95% HDI [−0.58, 0.57]). Detection sensitivity between ATC inclusion conditions was similar for both sessions 1 (BF_NULL_ = 2.71, δ = − 0.15, 95% HDI [− 0.74, 0.44]) and 2 (BF_NULL_ = 3.10, δ = − 0.14, 95% HDI [− 0.59, 0.58]).

To test whether ATC inclusion benefitted detection sensitivity, a within-subjects Bayesian t-test was conducted that compared *A’* between ATC inclusion conditions during baseline. Analysis yielded moderate evidence in favor of the null hypothesis (BF_NULL_ = 4.95, δ = − 0.07, 95% HDI [− 0.40, 0.22]) indicating no differences in detection sensitivity between ATC inclusion conditions at baseline. That is, *A’* values for simUTD performed with ATC (*M = *0.58, *SD = *0.23) were similar to *A'* values for simUTD performed without ATC (*M = *0.55, *SD = *0.23).

**Fig. 9:**
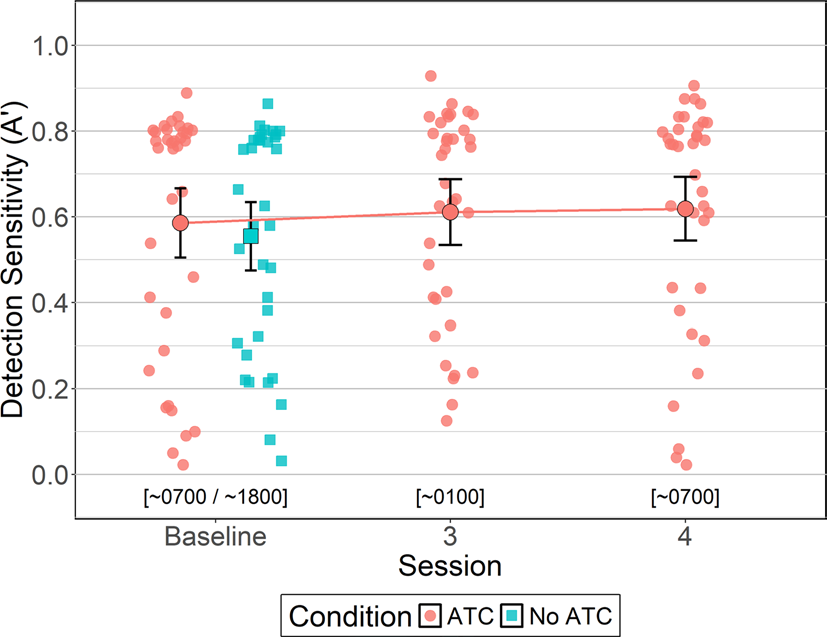
Detection Sensitivity (A’) by ATC Condition and Session. *Note* Error bars represent within-subject confidence intervals

A Bayesian ANOVA was conducted to evaluate whether ATC-included simUTD detection sensitivity changed as a function of fatigue. Bayesian ANOVA yielded moderate evidence in favor of the null hypothesis (BF_NULL_ = 9.89, *δ* = 0.29, 95% HDI [0.01, 0.86]) indicating that detection sensitivity did not differ across sessions. Baseline *A’* was 0.58 (*SD = *0.23), session 3 *A’* was 0.61 (*SD = *0.22), and session 4 *A’* was 0.61 (*SD = *0.21) (Fig. 9).

#### Detection Bias (B’’)

Between-subjects Bayesian t-tests were conducted to identify potential order effects for detection bias (i.e., *B''*, metacog which measures response tendency, where negative values indicate a liberal bias toward calling targets and positive values indicate a conservative bias toward rejecting targets). For No ATC, detection bias was similarly negative for sessions 1 (*M = *− 0.45, *SD = *0.51) and 2 (*M = *− 0.51, *SD = *0.29, BF_NULL_ = 2.96, *δ* = 0.08, 95% HDI [− 0.48, 0.68]) indicating liberal detection criterion across sessions. Likewise for ATC included, detection bias was similarly negative for sessions 1 (*M = *− 0.76, *SD = *0.27) and 2 (*M = *− 0.50, *SD = *0.64, BF_NULL_ = 1.15, δ = − 0.42, 95% HDI [− 1.01, 0.20]). Detection bias between ATC inclusion conditions did not differ for sessions 1 (BF_ALT_ = 1.45, δ = −0.54, 95% HDI [− 0.04, 1.22]) and 2 (BF_NULL_ = 3.05, δ = − 0.05, 95% HDI [− 0.64, 0.51]).

To understand whether ATC inclusion introduced a general bias to detection criterion, a within-subjects Bayesian t-test was conducted that compared *B’’* between ATC inclusion conditions at baseline. Analysis yielded moderate evidence in favor of the null hypothesis (BF_NULL_ = 3.24, *δ* = 0.16, 95% HDI [− 0.13, 0.49]) indicating ATC inclusion did not introduce a different bias to the already liberal detection criterion at baseline. That is, *B’’* values for simUTD performed with ATC (*M = *− 0.62, *SD = *0.51) were similar to *B'’* values for simUTD performed without ATC (*M = *− 0.48, *SD = *0.42).

**Fig. 10:**
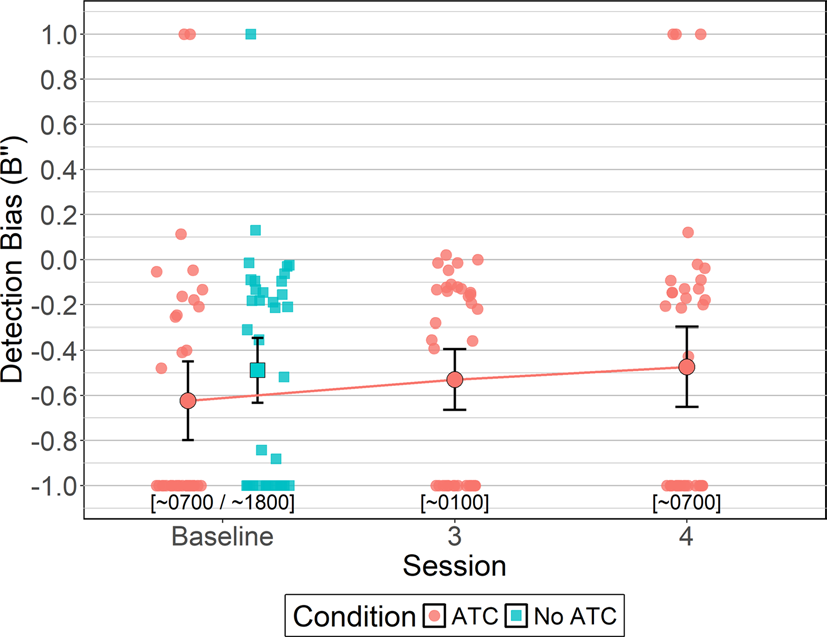
Detection Bias (B’’) by ATC Condition and Session. *Note* Detection bias (B’’) values equal to 0 indicate no bias, positive values indicate conservative bias, and negative values indicate liberal bias. Error bars represent within-subject confidence intervals

A Bayesian ANOVA was conducted to evaluate whether ATC-included simUTD detection bias changed as a function of fatigue. Bayesian ANOVA yielded moderate evidence in favor of the null hypothesis (BF_NULL_ = 5.80, *δ* = 0.32, 95% HDI [0.01, 0.94]) indicating that a general liberal bias was maintained across sessions. Baseline *B’’* was −0.62 (*SD = *0.51), session 3 *B’’* was −0.52 (*SD = *0.39), and session 4 *B’’* was − 0.47 (*SD = *0.52) (Fig. 10).

#### ATC Disuse and Misuse

Between-subjects Bayesian t-tests were conducted to separately identify potential order effects for ATC disuse (i.e., underutilization of the ATC when its cues are valid, measured as the percentage of correct ATC cues that were rejected) and misuse (overreliance on the ATC when its cues are invalid, measured as the percentage of incorrect ATC cues that were accepted). For disuse, analysis yielded moderate evidence in favor of the null hypothesis indicating disuse was similar between sessions 1 (*M = *20.83%, *SD = *5.2%) and 2 (*M = *22.22%, *SD = *6.1%, BF_NULL_ = 3.09, *δ *= − 0.034, 95% HDI [− 0.60, 0.55]). For misuse, analysis yielded weak evidence in favor of the null hypothesis indicating no differences between sessions 1 (*M = *30.94%, *SD = *2.6%) and 2 (*M = *22.70%, *SD = *1.9%, BF_NULL_ = 1.15, *δ* = 0.43, 95% HDI [− 0.18, 1.05]).

Separate Bayesian analysis of variances (ANOVAs) were conducted to evaluate whether disuse and misuse of the ATC changed as a function of fatigue. The Bayesian ANOVA evaluating disuse yielded moderate evidence in favor of the null hypothesis (BF_NULL_ = 9.67, *δ* = 0.29, 95% HDI [0.01, 0.89]) indicating that disuse did not differ across sessions. Baseline disuse was 21.7% (*SD = *24.62), session 3 disuse was 17.36% (*SD = *19.44), and session 4 disuse was 19.44% (*SD = *25.72).

**Fig. 11:**
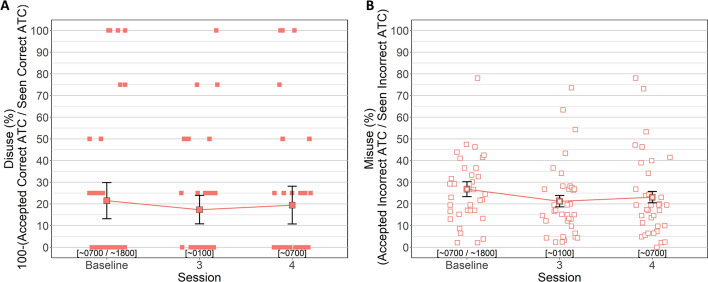
ATC Disuse and Misuse by Session. *Note.* **A** Disuse as a function of session. Higher values indicate greater underutilization of the ATC, or disuse. **B** Misuse as a function of session. Higher values indicate greater overreliance on the ATC, or misuse. Error bars represent within-subject confidence intervals

The Bayesian ANOVA evaluating misuse yielded weak evidence in favor of the alternative hypothesis (BF_ALT_ = 1.01, *δ* = 0.41, 95% HDI [0.02, 1.25]) indicating misuse did not differ across sessions. Baseline misuse was 26.82% (*SD = *1.6%), session 3 misuse was 21.24% (*SD = *1.2%), and session 4 misuse was 23.11% (*SD = *1.2%) (Fig. 11).

#### Metacognitive Sensitivity (AUROC2)

Between-subjects Bayesian *t*-tests were conducted to identify potential baseline session (i.e., session 1 vs. session 2) differences in metacognitive sensitivity (i.e., AUROC2, which measures how well confidence judgments track actual performance accuracy, with higher values indicating better alignment between confidence and accuracy) between and within ATC inclusion conditions (i.e., No ATC vs. ATC). For No ATC, metacognitive sensitivity was similar between sessions 1 (*M = *0.73, *SD = *0.06) and 2 (*M = *0.75, *SD = *0.09, BF_NULL_ = 2.89, δ = − 0.11, 95% HDI [− 0.70, 0.45]). Likewise for ATC-included simUTD, metacognitive sensitivity was similar between sessions 1 (*M = *0.78, *SD = *0.07) and 2 (*M = *0.71, *SD = *0.07, BF_ALT_ = 1.74, *δ* = 0.57, 95% HDI [− 0.06, 1.20]). Results also showed no differences between ATC inclusion conditions for session 1 (BF_NULL_ = 1.23, *δ* = − 0.41, 95% HDI [− 1.05, 0.16]) and session 2 (BF_NULL_ = 2.28, *δ* = 0.23, 95% HDI [− 0.32, 0.84]).

To test whether ATC inclusion benefitted metacognitive sensitivity, a within-subjects Bayesian t-test was conducted that compared metacognitive sensitivity between ATC inclusion conditions during the baseline session. Analysis yielded moderate evidence in favor of the null hypothesis (BF_NULL_ = 5.36, δ = − 0.44, 95% HDI [− 0.35, 0.27]) indicating no differences in metacognitive sensitivity between ATC inclusion conditions at baseline. That is, metacognitive sensitivity for simUTD performed with ATC (*M = *0.75, *SD = *0.07) was similar to simUTD performed without ATC (*M = *0.74, *SD = *0.08).

**Fig. 12:**
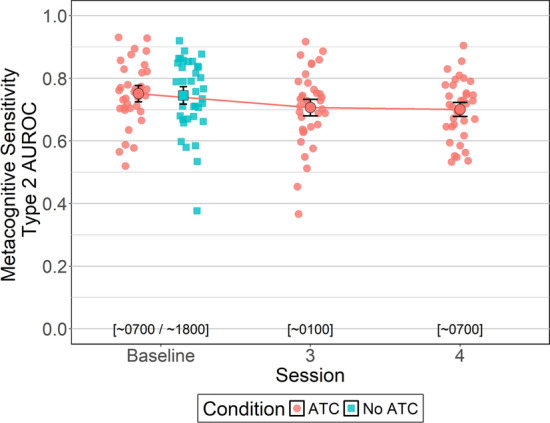
Metacognitive Sensitivity (type-2 AUROC) by ATC Condition and Session. *Note* Error bars represent within-subject confidence intervals

A Bayesian ANOVA was conducted to evaluate whether ATC-included simUTD metacognitive sensitivity changed as a function of fatigue. Bayesian ANOVA yielded moderate evidence in favor of the alternative hypothesis (BF_ALT_ = 3.26, *δ* = 0.47, 95% HDI [0.02, 1.54]) indicating that metacognitive sensitivity differed across sessions. Pairwise within-subjects Bayesian *t*-tests were performed were conducted between sessions (baseline vs. session 3 vs. session 4) to identify the pattern of change as a function of fatigue. Metacognitive sensitivity was similar between baseline and session 3 (*M = *0.71, *SD = *0.07, BF_ALT_ = 1.63, δ = 0.34, 95% HDI [0.01, 0.67]). However, there was moderate evidence that metacognitive sensitivity was greater at baseline than session 4 (*M = *0.70, *SD = *0.06, BF_ALT_ = 4.95, δ = 0.43, 95% HDI [0.10, 0.77]). Metacognitive sensitivity was similar between sessions 3 and 4 (BF_NULL_ = 5.27, *δ* = 0.05, 95% HDI [− 0.25, 0.38]) (Fig. 12).

#### Discrimination (Hit vs. FA Confidence Ratings)

Two Bayesian t-tests were conducted to evaluate differences in confidence ratings between hits and FAs within ATC inclusion conditions (i.e., No ATC vs. ATC) at baseline. For ATC simUTD, participants had higher confidence for hits (*M = *3.65, *SD = *0.60) than FAs (*M = *2.11, *SD = *0.22, BF_ALT_ > 100,000, *δ* = 2.22, 95% HDI [1.62, 2.84]). Likewise for No ATC simUTD, participants had higher confidence for hits (*M = *3.62, *SD = *0.51) than FAs (*M = *2.17, *SD = *0.38, BF_ALT_ > 100,000, *δ* = 1.94, 95% HDI [1.39, 2.55]). Additional Bayesian t-tests were conducted to test whether there were differences in hit or FA confidence ratings between ATC inclusion conditions at baseline. This analysis showed confidence ratings for hits were similar between ATC and no ATC conditions (BF_NULL_ = 3.98, *δ* = 0.03, 95% HDI [− 0.39, 0.48]). Confidence ratings for FAs were also similar between ATC and no ATC conditions (BF_NULL_ = 3.79, *δ* = − 0.09, 95% HDI [− 0.54, 0.32]).

A Bayesian ANOVA was conducted to evaluate changes in confidence ratings between hits and FAs as a function of fatigue. Bayesian ANOVA showed extreme evidence in favor of the alternative hypotheses for the signal detection variable (hits vs. FAs) main effect model (BF_ALT_ > 100,000, δ = 20.88, 95% HDI [0.17, 30.75]), the session main effect model (BF_ALT_ > 100,000, *δ* = 0.28, 95% HDI [0.01, 0.82]), and the signal detection variable X session interaction model (BF_ALT_ > 100,000, *δ* = 0.36, 95% HDI [0.02, 1.09]).

**Fig. 13:**
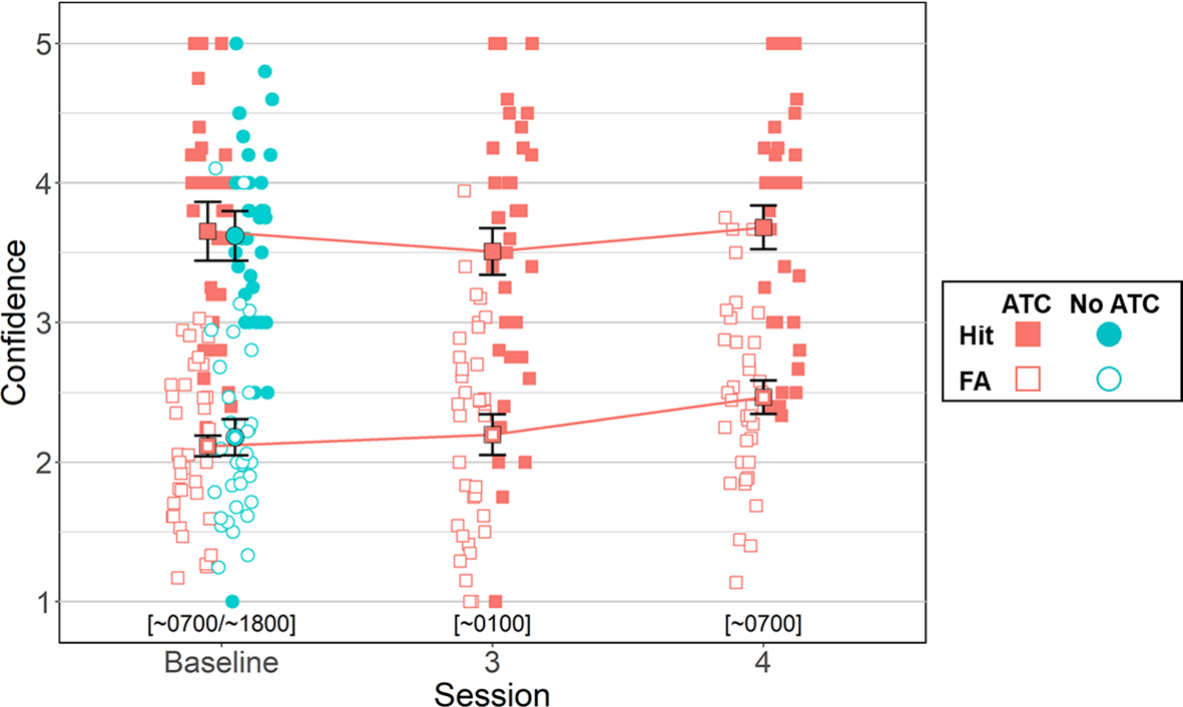
Hit versus False Alarm Average Confidence Ratings by ATC Condition and Session. *Note.* Error bars represent within-subject confidence intervals

Overall confidence ratings for hits (*M = *3.61, *SD = *0.88) were higher than confidence ratings for FAs (*M = *2.26, *SD = *0.66, BF_ALT_ > 100,000, *δ* = 1.69, 95% HDI [1.37, 2.00]). Comparing confidence ratings across sessions for hits only yielded moderate evidence in favor of the null hypothesis between all sessions indicating average confidence ratings for hits were similar across sessions (baseline vs. session 3: BF_NULL_ = 3.33, *δ* = 0.14, 95% HDI [− 0.30, 0.59]; baseline vs. session 4: BF_NULL_ = 3.95, δ = − 0.03, 95% HDI [− 0.49, 0.39]; session 3 versus 4: BF_NULL_ = 3.15, δ = − 0.15, 95% HDI [− 0.60, 0.28]. The same comparison across sessions for FAs only showed no differences in confidence ratings between baseline (*M = *2.11, *SD = *.22) and session 3 (*M = *2.19, *SD = *0.07, BF_NULL_ = 3.66, *δ* = − 0.10, 95% HDI [− 0.53, 0.32]) as well as no differences between sessions 3 and 4 (*M = *2.46, *SD = *0.35, BF_NULL_ = 1.34, δ = − 0.33, 95% HDI [− 0.77, 0.10]). However, confidence ratings for FAs were higher at session 4 than baseline (BF_ALT_ = 3.21, δ = − 0.51, 95% HDI [− 0.99, − 0.08]) (Fig. 13).

#### Trust in the ATC

**Fig. 14:**
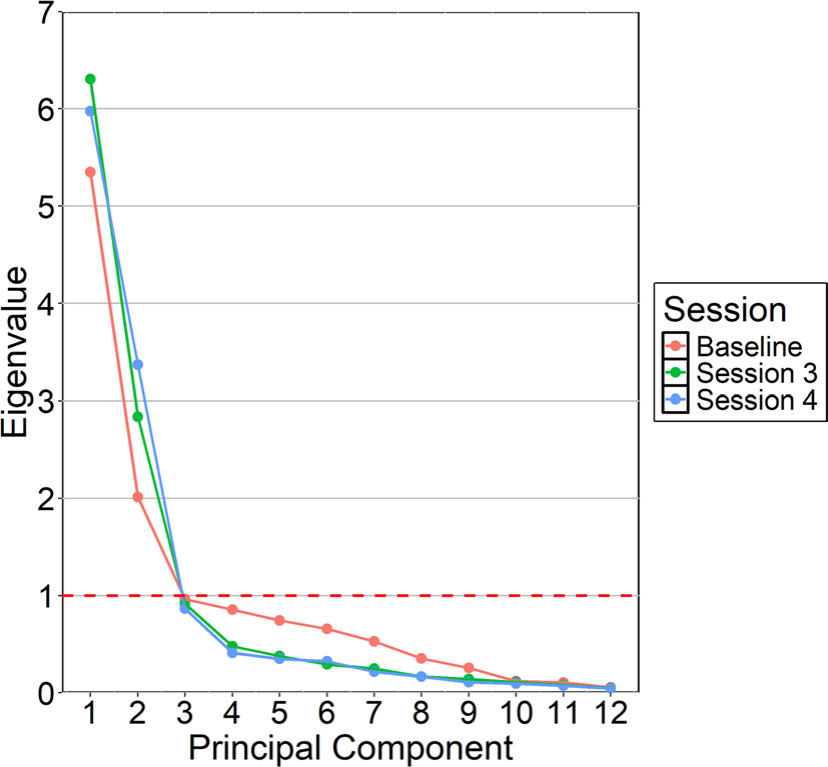
Scree Plot of Principal Component Eigenvalues for Each Session. *Note* Red dashed horizontal line depicts Kaiser criterion threshold for principal component retention (Kaiser, 1960)

Principal components analysis (PCA) was conducted to investigate whether a) the first two principal components (PC) reflect two separate trust and distrust constructs as originally demonstrated by Jian and colleagues’ (2000) and b) the number of principal components and their loadings stay consistent as a function of fatigue. Three PCAs were conducted, one per session (i.e., baseline vs. session 3 vs. session 4). Only PCs with eigenvalues greater than one were extracted (See Figure 14; Kaiser, 1960). The baseline session PCA revealed the first two PCs accounted for 62% of the total variance. Session 3 PCA revealed the first two PCs accounted for 76% of the total variance. Session 4 PCA revealed the first two PCs accounted for 78% of the variance.

Table 1 shows the loadings of the trust in ATC items on the first two PCs for each session. Across all sessions, PC_1_ captures the construct of positive trust with perceptions of security, integrity, dependability, reliability, and trust being the highest loading items. Of note, however, confidence in the ATC was not a high loading item for PC_1_ at baseline, though it became a high loading item for the subsequent sessions. PC_2_ on the other hand generally captured negative trust (or distrust) with perceptions of deceptiveness, underhandedness, and suspicion being the highest loading items across all sessions. Additionally for sessions 3 and 4 perceptions of wariness and harmfulness of the ATC were also high loadings for PC_2_. Unsurprisingly, perceptions of familiarity did not have high loadings for either of the first two PCs across sessions which aligns with previous research suggesting that familiarity is a unique component of trust that influences the trust relationship though it is treated separately from concepts that underly positive and negative trust.

**Table 1:** Varimax Rotated Principal Component Loadings for Trust in ATC Items across Sessions

Trust in ATC item	Baseline		Session 3		session 4	
	PC_1_	PC_2_	PC_1_	PC_2_	PC_1_	PC_2_
Is deceptive?	− 0.06	***0.78***	− 0.09	***0.91***	− 0.07	***0.91***
Is underhanded?	0.03	***0.79***	− 0.02	***0.91***	− 0.07	***0.95***
Am suspicious of?	− 0.21	***0.83***	− 0.21	***0.9***	− 0.03	***0.93***
Am wary of?	− 0.07	0.63	− 0.17	***0.85***	− 0.17	***0.88***
Is harmful?	− 0.3	0.53	− 0.23	***0.76***	− 0.2	***0.86***
Am confident in?	0.52	− 0.5	***0.85***	− 0.24	***0.9***	− 0.16
Provides security?	***0.84***	0.06	***0.92***	− 0.08	***0.88***	0.07
Has integrity?	***0.7***	− 0.17	***0.82***	− 0.16	***0.82***	− 0.13
Is dependable?	***0.74***	− 0.52	***0.91***	− 0.27	***0.89***	− 0.2
Is reliable?	***0.78***	− 0.51	***0.91***	− 0.23	***0.93***	− 0.21
Is trustworthy?	***0.79***	− 0.34	***0.92***	− 0.22	***0.89***	− 0.28
Am familiar with?	0.66	0.17	0.47	0.04	0.48	0.06

Bolded and italic values highlight highest loadings ($\ge .7$) for each principal component*.*

PCA analysis revealed relatively consistent loadings for the first two PCs across sessions; separate trust and distrust scores were calculated by averaging the highest loading items for trust (i.e., perceptions of confidence, security, integrity, dependability, reliability, and trustworthiness) and distrust (i.e., perception of deceptiveness, underhandedness, suspiciousness, wariness, and harmfulness) separately. Moreover, familiarity did not load on either the trust or distrust components suggesting that familiarity is treated as a separate construct. Three Bayesian ANOVAs were conducted, one with distrust, one with trust, and one with familiarity as the dependent variables, to evaluate whether distrust, trust, and familiarity changed as a function of fatigue.

**Fig. 15:**
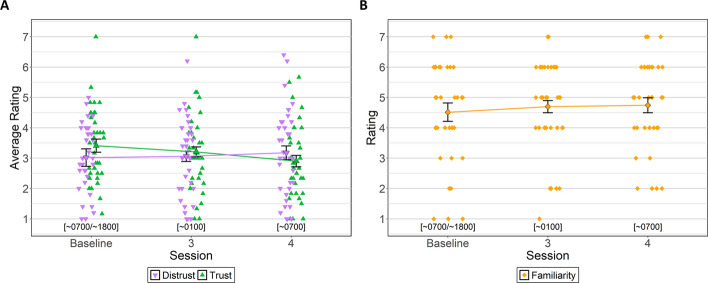
Trust, Distrust, and Familiarity Ratings by Session. *Note* **A** Trust and distrust in the ATC average ratings by session. **B** Familiarity with the ATC ratings by session. Error bars represent within-subject confidence intervals

Bayesian ANOVA for distrust yielded moderate evidence in favor of the alternative hypothesis (BF_NULL_ = 6.3, *δ* = 0.35, 95% HDI [0.02, 0.99]) suggesting perceptions of distrust in the ATC did not change across session. Alternatively, Bayesian ANOVA for trust yielded strong evidence in favor of the alternative hypothesis (BF_ALT_ = 30.97, *δ* = 0.62, 95% HDI [0.02, 1.90]) suggesting perceptions of trust in the ATC changed across session. Pairwise within-subjects Bayesian t-tests were conducted between sessions (baseline vs. session 3 vs. session 4) to identify the pattern of change in perceptions of trust as a function of session. These analyses revealed no differences in trust in the ATC between baseline (*M = *3.40, *SD = *1.19) and session 3 (*M = *3.20, *SD = *1.26, BF_NULL_ = 1.40, δ = 0.28, 95% HDI [− 0.08, 0.61]). However, when baseline and session 4 were compared, there was strong evidence in favor of the alternative hypothesis (BF_ALT_ = 25.43, δ = 0.57, 95% HDI [0.20, 0.94]) indicating participants had higher trust in the ATC at baseline than session 4 (*M = *2.91, *SD = *1.20). There was also moderate evidence that trust was higher at session 3 when compared to session 4 (BF_ALT_ = 3.12, δ = 0.42, 95% HDI [0.06, 0.77]). Finally, Bayesian ANOVA for familiarity yielded moderate evidence in favor of the null hypothesis (BF_NULL_ = 4.28, *δ* = 0.33, 95% HDI [0.02, 1.00]) indicating perceptions of familiarity with the ATC did not change across session (Fig. 15).

### Discussion

The present study investigated the utility of integrating a low LOA ATC system into the UTD task workflow. We primarily sought to evaluate whether target detection performance and metacognitive judgements (i.e., confidence in calls and trust) benefitted from the inclusion of ATC, especially under conditions of increased fatigue due to time awake. Critically, the ATC behavior was imperfect and did not change across sessions; therefore, any differences across session reflect the influence of increased fatigue. We did not find that performance (broadly) benefitted from ATC. Instead, there was evidence suggesting ATC slowed task completion and increased false alarm rate initially. These findings suggest that including ATC in the simUTD task workflow does not enhance detection performance when participants are in a relatively high alert state. Moreover, disuse and misuse did not change as a function of fatigue. At baseline, metacognitive sensitivity and discrimination were similar between ATC inclusion conditions. However, metacognitive sensitivity was worse at session 4 compared to baseline driven by inflated confidence in false alarm calls at session 4. It appears that the mere inclusion of ATC also does not incidentally enhance metacognitive judgements.

We generally replicated Jian and colleagues’ (2000) findings that trust and distrust could be treated as two separate constructs. The trust construct forms with perceptions of confidence, security, dependability, and reliability, while perceptions of wariness, suspiciousness, deceptiveness, underhandedness, and harmfulness form the distrust construct. However, baseline perceptions of confidence did not load on the trust component and perceptions of wariness and suspiciousness did not load on the distrust component. This finding indicates that participants considered these concepts differently after initial interaction with the ATC compared to their subsequent interaction under progressive fatigue. After determining trust and distrust could be evaluated separately, we observed trust in the ATC decreased with fatigue while perceptions of distrust did not change. Perceived familiarity with the ATC also did not load on either trust and distrust, indicating familiarity judgments were considered independently of trust and distrust. It is noteworthy that perceptions of familiarity with the ATC did not change across sessions further supporting the notion that the observed changes in metacognitive sensitivity and trust can be attributed to increased fatigue and not repeated use with the consistent, yet imperfect ATC.

#### Benefits and Costs of Automation Assistance under Fatigue

Though ATC inclusion did not improve detection performance and metacognition under low fatigue conditions, we were equally interested in evaluating performance patterns across increasing levels of fatigue. Participants maintained relatively high levels of performance as time awake increased. Detection sensitivity remained consistently moderate (~0.6 across sessions), driven by relatively high hit rates (~80%) maintained across sessions, and high FA rates that reduced slightly from baseline (~80%) to the latter sessions (~72%). However, due to our study design, we cannot directly attribute this maintained performance to ATC assistance, as we did not include No ATC sessions during periods of high fatigue. Detection bias also remained consistent across low and high fatigue sessions, with a majority of participants generally favoring a liberal detection bias largely driven by parity between FA and hit rates. It is important to note that in addition to not observing performance degradation as a function of fatigue, we also did not observe performance degradation during session 3 (0100) when alertness dropped considerably (See Figure 3). Previous research has shown performance tends suffer after ~16 h of wakefulness (Killgore, 2010). While it is tempting to attribute these results as demonstrating use of ATC is protective against fatigue-related vigilance and performance degradation, we have previously established that participants maintained high simUTD task performance during sleep restriction without ATC (Dunn et al., 2023, Goel et al., 2013; Killgore, 2010). It is noteworthy that participants in both studies were screened to have completed a basic training course and reported having operational experience performing UTD. Therefore, we favor the interpretation that task expertise as well as learning optimizing target visual search, rather than ATC assistance, most likely accounted for the maintained high detection performance despite fatigue.

We furthermore replicated our previous result showing that as time awake increased, total task time doing simUTD decreased (Dunn et al., 2023). Alertness scores aligned with established circadian patterns, remaining relatively high from morning to early evening, dropping sharply in the late evening, stabilizing at a low point before recovering slightly prior to normal wake time (i.e., circadian rescue, See Sheldon et al., 2014). Despite a subtle increase in alertness scores prior to simUTD 4 (See Figure 4), participants perceived levels of sleepiness monotonically increased throughout the wakeful period (See Figure 5). Thus, it is possible that even though participants felt progressively sleepier, the observed decrease in total task time reflects an adaptation to the task demands or greater motivation to complete the task sooner. Previous research has shown that task repetition benefits visual search response time, especially when the target does not change across multiple searches (Ahissar & Hochstein, 2004; Wolfe et al., 2000). Though the primary target object type, varied from session to session, all object types were present across all sessions. Moreover, the general defining features of the three target objects were similar (i.e., sonar shadow cast by the object and size). Task difficulty, search context (i.e., ocean bottom features), and ATC behavior (probability of detection and FA rate) also remained consistent throughout the sessions. It is therefore not surprising, yet still notable, that despite fatigue participants were progressively faster to complete the simUTD without compromising search performance.

Interestingly, metacognitive sensitivity reduced between baseline and sessions 3 and 4. As noted, analysis of metacognitive discrimination showed that the reduction in metacognitive sensitivity could be attributed to an increase in confidence in FA calls as a function of fatigue since confidence in hits remained stable across sessions. Previous research has shown mild prefrontal lobe dysfunction due to even acute sleep deprivation (< 24 h wakefulness) can produce discrepancies between task low-level and higher-order cognitive and attentional processes (Aidman et al., 2019; Jackson & Kleitman, 2014; Killgore et al., 2007). As shown in a meta-analysis by Lim and Dinges (2010), performance on orienting or executive attention tasks, akin to the simUTD task, remains relatively preserved despite sleep deprivation. However, sleep loss negatively affects a host of higher-order attentional and cognitive processes that require supervisory control and task monitoring, such as working memory (Smith et al., 2002), vigilance (Harrison & Horne, 2000; Killgore, 2010), problem-solving (Linde & Bergstrom, 1992), inhibitory control (Drummond et al., 2006), and complex decision-making (Harrison et al., 2007; Harrison & Horne, 1999; Killgore et al., 2006; Linde et al., 1999), including metacognitive judgements (Bard et al., 1996; Harrison & Horne, 2000).

#### Fatigue Negatively Impacts Trust in Automation

Evaluating the utility of an assistive technology, such as ATC, entails determining whether the user perceives it as a useful and effective tool. The ATC in this study was programmed to be good yet imperfect, mimicking the performance of other machine learning-based image recognition detection aid tools. Use behavior of the ATC (i.e., Disuse and Misuse) was decently calibrated on average and consistent with increased fatigue. That is, disuse was relatively low (~20%) across sessions indicating that there was a high propensity to accept ATC calls that were valid. Misuse was also relatively low (~25%) across sessions indicating a low propensity to accept ATC calls that were invalid. Given the ATC was good, yet imperfect, and participants had to rely on their experience with the ATC to determine its reliability, our results suggest that participants a) made use of the ATC cues when performing the task and b) maintained a reasonable level of skepticism required to avoid underutilization and overreliance. However, use was not perfectly calibrated indicating room for improvement. For example, previous research has shown that transparency about the reliability of the automation can improve performance and use (Mercado et al., 2016). Moreover, though distrust and familiarity did not change across sessions, trust decreased.

Together, these findings further demonstrate that fatigue may uniquely impact higher-order processes mediated by regions of the PFC critical for making metacognitive judgements, including evaluating trust (Harrision & Horne, 2000; Hopko & Mehta, 2024; Parasuraman et al., 2014; Perello-March et al., 2022). It is worthwhile to note that FA rate and use were unaffected by the imperfect ATC, which have previously been attributed to increased automation FA rate (Krupinski et al., 1993). FA rates were high, but similar to FA rates in our previous study where participants conducted simUTD without ATC (Dunn et al., 2023). Our findings provide key insights that contribute to a more nuanced view of how fatigue impacts trust in automation. It has previously been shown that fatigue can negatively impact trust in automation, leading to higher rejection rates of automated assistance (Schaefer et al., 2016). However, participants in the present study did not reject valid and invalid cues at higher rates as a function of fatigue. Rather, task performance and use of the ATC were unaffected by fatigue, while metacognitive sensitivity and trust in the ATC decreased. Thus, it appears that fatigue may have somewhat dissociative effects, negatively impacting higher-order decision-making while preserving lower-order visual detection so long as an adequate level of arousal is maintained to make visual detections.

#### Limitations

Several limitations should be considered when interpreting these findings. First, although each participants completed simUTD sessions with and without ATC during periods of high alertness (sessions 1 and 2), they did not complete simUTD without ATC during the low alertness period (sessions 3 and 4). While an alternative design comparing ATC vs. No ATC conditions during high fatigue sessions would have been methodologically ideal, the practical constraints of working with this specialized military population necessitated our chosen design. The operators were available during a limited training period, making it unfeasible to conduct multiple overnight studies with the same participants. However, this approach allowed us to maximize our sample of operators with the specialized expertise required for this task, while still gathering valuable data about performance patterns with ATC under increased fatigue. To help interpret these patterns, we reference our previous research which employed a similar time awake (~24 h) protocol for evaluating the effects of fatigue on simUTD task performance without ATC for comparison (Dunn et al., 2023).

Second, the ATC was programmed to reflect the imperfections and capabilities of fielded systems, though there are different ways in which an ATC system can be imperfect beyond varying the probability of detection and false alarm rates. For example, the type of false alarms may matter as well. If the ATC consistently calls objects dissimilar to the target objects (i.e., a rock, fish trap, tire, or debris), false positive calls may challenge operator trust and ATC usage to a greater extent than calling objects that share perceptual features with target objects. Moreover, we did not provide participants with ATC confidence values associated with each call. It is still an open question whether providing this information is beneficial to participants using the ATC. McLeod et al. (2005) found that trust in automation is highly sensitive to system reliability. Additionally, de Visser and Parasuraman (2011) have emphasized that even imperfect automation could be beneficial if operators understand its limitations. Therefore, the presence of ATC confidence values may aid performance by providing information relevant to higher-order metacognitive judgments concerning confidence in detection accuracy as well as trust in the automation.

#### Practical Implications and Future Directions

These findings have several practical implications for the design and implementation of ATC systems in military settings, as well as other professions that require high-stakes prolonged visual search (e.g., mine countermeasures, aerial reconnaissance, search and rescue, etc.). The stability of detection performance across fatigue conditions is promising, though we were hesitant to attribute maintenance of detection sensitivity to the inclusion of ATC considering high operator experience. Nonetheless, it is clear that ATC did not harm detection performance, and participants quickly adapted to the ATC despite fatigue as reflected in the decreased total task time.

The observed decline in metacognition and trust shows that simple visual cuing may not facilitate optimal calibration to the ATC system. Therefore, it may be useful to provide ATC confidence information in addition to the simple cue to aid operators in achieving calibrated use, especially when confidence judgments are an integral component to overall task performance. Such information may also benefit overall trust in the ATC by not only by providing information that directly inform metacognitive decisions, but it also promotes greater transparency (Hou et al., 2021).

### Conclusions

Decisions made in military contexts have profound consequences. Thus, it is critical to understand the factors that bias these factors for better or worse. The UTD example investigated here highlights the immense challenges operators face when making real-world life or death decisions. The rapid development of technologies that have the potential to provide much needed aid in these contexts shows promise. However, as shown here, the anticipated potential of integrating automation does not always produce the desired benefit. We found participants used the ATC when it was included and that use behavior was reasonably calibrated and did not change as a function of fatigue. Critically, however, we found that ATC did not enhance target detection and classification performance relative to not having it during high alertness times of the day, though our results did show that detection performance with ATC did not degrade with fatigue. Of note, metacognitive sensitivity reduced with fatigue driven by higher confidence in false alarms. This is worrisome given sleep deprivation is a persistent problem in the military and the weight of confidence judgements in performing safety critical tasks. Finally, we were able to replicate Jian and colleagues’ (2000) findings that trust and distrust can be treated as separable constructs independent of familiarity. This exploratory analysis revealed trust decreased with fatigue and/or use while distrust maintained.

## Supplementary Information

Supplementary file 1.

## Data Availability

The datasets generated and/or analyzed during the current study are not publicly available due to security protocols and privacy regulations, but they may be made available on reasonable request by the Naval Health Research Center Institutional Review Board (contact phone 619-553-8400)

## Abbreviations

ATC: Automatic target cuing
FA: False alarm
KSS: Karolinska Sleepiness Scale
PCA: Principal components analysis
UTD: Undersea threat detection
