# Supplementary material for: Dissociable effects of fatigue on performance and metacognition from automatic target cuing in undersea threat detection
Source: Cogn Res Princ Implic. 2025 Jun 15;10:29. doi: 10.1186/s41235-025-00638-1 (PMC12167736; doi:10.1186/s41235-025-00638-1)
Supplement: Supplementary file 1 [file 41235_2025_638_MOESM1_ESM.pdf]

Supplemental Materials

Checklist for Trust in the ATC (Adapted from Jian et al., 2000)

Below is a list of statement for evaluating trust between people and automation. There are scales for you to rate intensity of your feeling of trust, or your impression of the Automatic Target Cuing (ATC) system after completing the simulated undersea threat detection task. Please circle on the number which best describes your feeling or your impression.

Note: Not at all = 1; Extremely = 7

1. The ATC system is deceptive

1 2 3 4 5 6 7

2. The ATC system behaves in an underhanded manner

1 2 3 4 5 6 7

3. I am suspicious of the ATC system's intent, action, or outputs

1 2 3 4 5 6 7

4. I am wary of the ATC system

1 2 3 4 5 6 7

5. The ATC system's actions will have a harmful or injurious outcome

1 2 3 4 5 6 7

6. I am confident in the ATC system

1 2 3 4 5 6 7

7. The ATC system provides security

1 2 3 4 5 6 7

8. The ATC system has integrity

1 2 3 4 5 6 7

9. The ATC system is dependable

1 2 3 4 5 6 7

10. The ATC system is reliable

1 2 3 4 5 6 7

11. I can trust the ATC system

1 2 3 4 5 6 7

12. I am familiar with the ATC system

1 2 3 4 5 6 7
